# Primary adhered neutrophils increase JNK1-MARCKSL1-mediated filopodia to promote secondary neutrophil transmigration

**DOI:** 10.1016/j.isci.2023.107406

**Published:** 2023-07-17

**Authors:** Max Laurens Bastiaan Grönloh, Janine Johanna Geertruida Arts, Eike Karin Mahlandt, Martijn A. Nolte, Joachim Goedhart, Jaap Diederik van Buul

**Affiliations:** 1 Vascular Biology Lab, Medical Biochemistry Department at Amsterdam UMC, University of Amsterdam, Amsterdam, the Netherlands; 2 Leeuwenhoek Centre for Advanced Microscopy, section Molecular Cytology at Swammerdam Institute for Life Sciences at the University of Amsterdam, Amsterdam, the Netherlands; 3 Molecular Cell Biology Lab at Department Molecular Hematology, Sanquin Research, and Landsteiner Laboratory, Amsterdam, the Netherlands

**Keywords:** Molecular biology, Immunology, Cell biology

## Abstract

During inflammation, leukocytes extravasate the vasculature to areas of inflammation in a process termed transendothelial migration. Previous research has shown that transendothelial migration hotspots exist, areas in the vasculature that are preferred by leukocytes to cross. Several factors that contribute to hotspot-mediated transmigration have been proposed already, but whether one leukocyte transmigration hotspot can be used subsequently by a second wave of leukocytes and thereby can increase the efficiency of leukocyte transmigration is not well understood. Here, we show that primary neutrophil adhesion to the endothelium triggers endothelial transmigration hotspots, allowing secondary neutrophils to cross the endothelium more efficiently. Mechanistically, we show that primary neutrophil adhesion increases the number of endothelial apical filopodia, resulting in an increase in the number of adherent secondary neutrophils. Using fluorescence resonance energy transfer (FRET)-based biosensors, we found that neutrophil adhesion did not trigger the activity of the small GTPase Cdc42. We used kinase translocation reporters to study the activity of mitogen-activated protein (MAP) kinases and Akt in endothelial cells on a single-cell level with a high temporal resolution during the process of leukocyte transmigration and found that c-Jun N-terminal kinase (JNK) is rapidly activated upon neutrophil adhesion, whereas extracellular regulated kinase (ERK), p38, and Akt are not. Additionally, we show that short-term chemical inhibition of endothelial JNK successfully prevents the adhesion of neutrophils to the endothelium. Furthermore, we show that neutrophil-induced endothelial JNK1 but not JNK2 increases the formation of filopodia and thereby the adhesion of secondary neutrophils. JNK1 needs its downstream substrate MARCKSL1 to trigger additional apical filopodia and consequently neutrophil adhesion. Overall, our data show that primary neutrophils can trigger the endothelial transmigration hotspot by activating JNK1 and MARCKSL1 to induce filopodia that trigger more neutrophils to transmigrate at the endothelial hotspot area.

## Highlights

• Neutrophil adhesion promotes secondary neutrophil adhesion by increasing filopodia
• Biosensors revealed only JNK, and no ERK/p38/Akt, activation upon neutrophil adhesion
• Apical filopodia are induced by a neutrophil-induced JNK1-MARCKSL1 pathway

## Graphical abstract

**Figure:**
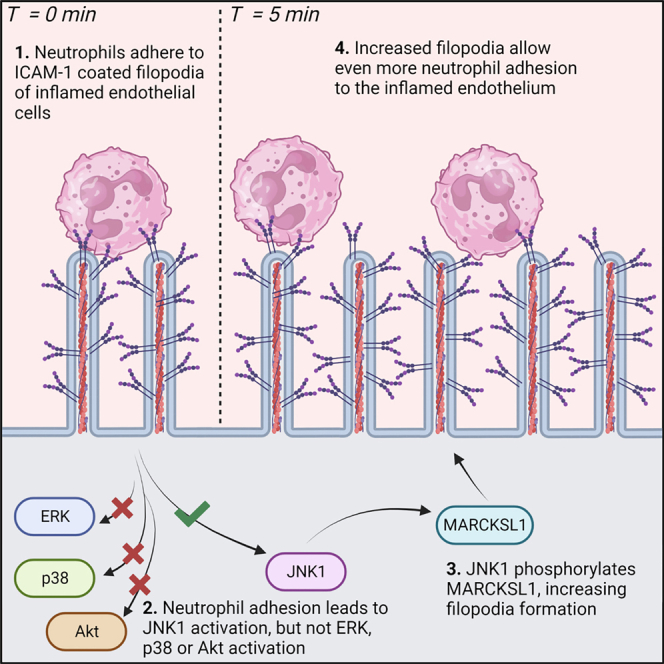

## Introduction

To reach sites of infection, leukocytes need to cross the endothelial monolayer in a process termed transendothelial migration (TEM). During TEM, leukocytes undergo a sequence of well-established steps: rolling, adhesion, crawling, diapedesis, and abluminal crawling.^1^^,^^2^^,^^3^^,^^4^^,^^5^ Moreover, it is established that leukocytes undergo transmigration not randomly but at defined hotspots.^6^^,^^7^^,^^8^ These hotspots can be regulated by the endothelial cell, for example, by heterogeneous adhesion molecule expression, chemokine gradients, substrate stiffness, or junctional membrane protrusions. Additionally, neutrophils can bind adherent neutrophils via L-selectin. By transmigrating at the same sites, the barrier integrity of the endothelial monolayer is preserved and leakage during diapedesis is minimized.^8^

TEM dynamics differ between vascular beds and leukocyte subsets but generally do not span more than a few minutes between the first contacts during rolling and the final crossing of the endothelial monolayer.^9^ During this short time, leukocyte-endothelial interactions result in many signaling processes that allow transmigration of leukocytes. For example, leukocyte integrins bind the endothelial intercellular adhesion molecule-1 (ICAM-1), causing ICAM-1 to cluster and trigger many downstream signal cascades via its intracellular tail.^10^^,^^11^ ICAM-1 is upregulated upon inflammation and localizes to apical filopodia extending into the vascular lumen, where these actin-based protrusions aid in the adhesion of leukocytes.^12^ These filopodia are believed to create a larger surface area for leukocytes to adhere to the endothelium and to encapsulate leukocytes from the circulation into a cup-like membrane structure before commencing into the later stages of TEM.^13^^,^^14^^,^^15^^,^^16^ However, although we have started to understand the initial signaling events preceding formation of apical filopodia, the details have not been fully understood yet.

Among the signaling pathways described downstream of leukocyte adhesion is the mitogen-activated protein kinase (MAPK) pathway, which consists of a cascade of kinases that are known to regulate a wide variety of biological processes.^17^^,^^18^ The three main protein subfamilies in the MAPK protein family are the extracellular regulated kinases (ERKs), c-Jun N-terminal kinases (JNKs), and p38. The MAPKs are most well-known for their transcription factor substrates and thus their gene expression effects. However, JNK also phosphorylates cytoplasmic, mitochondrial, cytoskeletal, and membrane-associated substrates that do not require gene expression directly and are thus able to facilitate rapid intracellular signaling.^19^^,^^20^ In the context of TEM, previous studies have demonstrated, based on biochemical phosphorylation assays, the activation of ERK, JNK, and p38 downstream of ICAM-1 clustering and subsequently linked JNK activation to the phosphorylation of paxillin and internalization of vascular endothelial cadherin (VE-cadherin).^21^

Up until recently, activation of kinases was primarily studied through biochemical assays, measuring the phosphorylation status of a kinase from total cell lysates. Though these studies provided valuable insights in the molecular pathways, these did not tell us when and where these kinases are activated on a single-cell level. The development of synthetic kinase translocation reporters (KTRs) allowed the field to study kinases on single-cell level with a high temporal resolution using microscopy-based assays.^22^ KTRs for ERK, p38, and JNK have been described and have been used to investigate their activation dynamics after different types of stimuli and conditions.^23^^,^^24^^,^^25^ How MAPKs are temporally regulated on a single-endothelial-cell level during leukocyte TEM is not known.

Here, we show that neutrophils increase the number of apical filopodia in tumor necrosis factor-alpha (TNFα)-treated endothelial cells (ECs) within minutes after initial adhesion, independently of Cdc42, a Rho GTPase known to be involved in TNFα-mediated filopodia formation.^12^ Instead, by using the KTR biosensors, we found that specifically JNK is activated in ECs upon initial neutrophil adhesion, whereas ERK, Akt, or p38 are not. Additionally, we show that short-term JNK inhibition leads to decreased neutrophil adhesion, and conversely, endothelial JNK1 overexpression increases adhesion. We link JNK signaling to the formation of apical filopodia, and our data suggest a positive feedback loop, in which neutrophil adhesion leads to enhanced JNK activation, in turn leading to an increase in the number of apical filopodia and consequently increased adhesion of a second batch of neutrophils. Finally, we show that myristoylated alanine-rich C kinase substrate-like 1 (MARCKSL1), a known JNK substrate, is responsible for neutrophil-induced apical filopodia formation and secondary neutrophil adhesion. Together, we show that neutrophils can prime TEM hotspots by increasing the number of filopodia through immediate JNK activation and MARCKSL1, resulting in enhanced neutrophil transmigration.

## Results

### Primary neutrophil adhesion promotes secondary neutrophil adhesion by increasing apical filopodia

**Figure 1:**
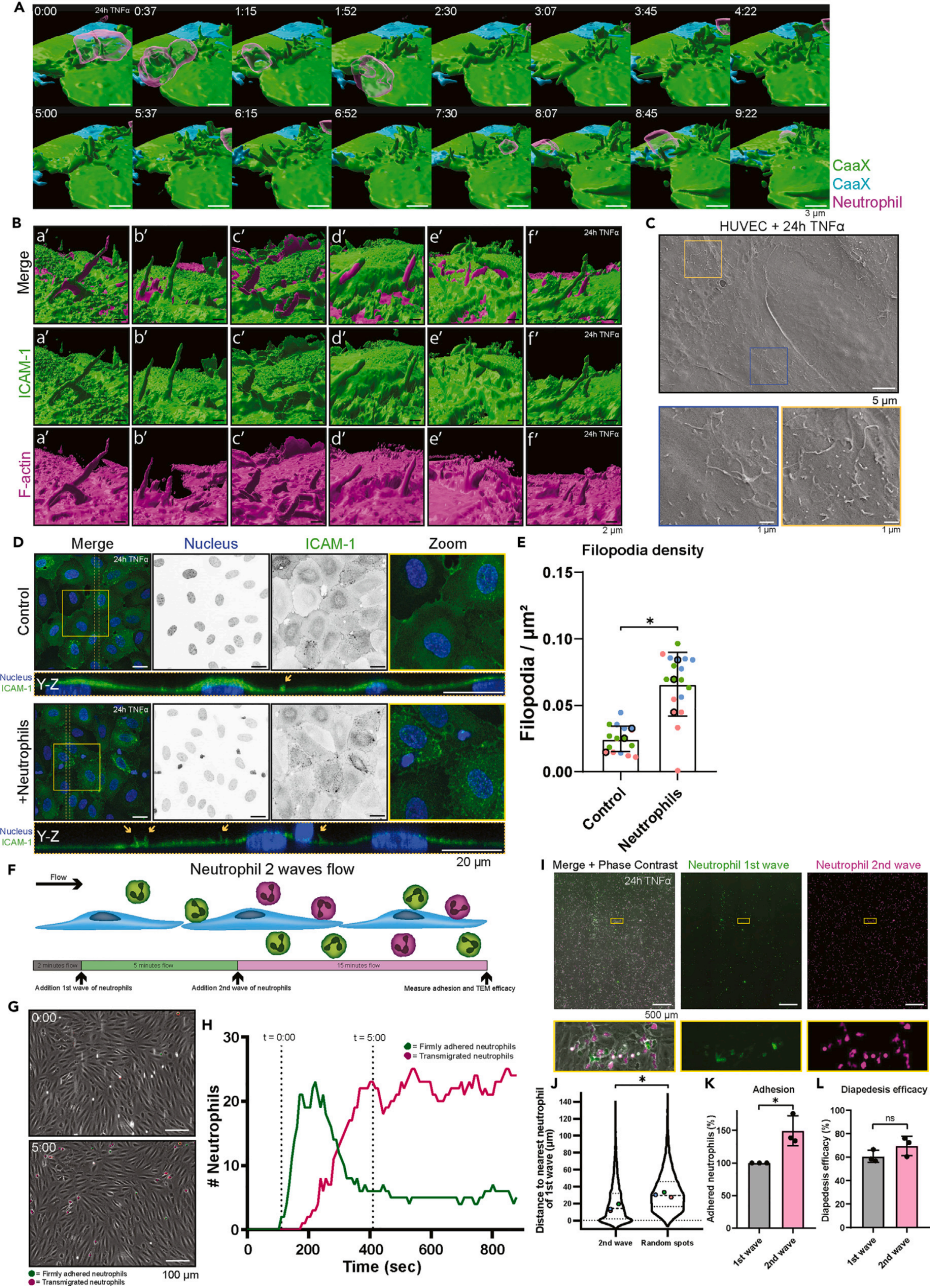
Neutrophil adhesion induces apical filopodia (A) Stills from a time-lapse video of neutrophils (magenta) migrating through 24-h TNFα-treated CaaX-transduced endothelial cells (green and cyan) and the dynamics of apical endothelial protrusions. Time is indicated in min:sec. Scale bar, 3 μm. (B) Different example stills from a time-lapse video of 24-h TNFα-treated endothelial cells co-transduced with ICAM-1 (green) and LifeAct (magenta). Scale bar, 2 μm. (C) Scanning electron microscopy image of 24-h TNFα-treated endothelial cells. Scale bar, 5 μm. Blue and orange rectangles show zooms. Scale bar, 1 μm. (D) Immunofluorescent stains of HUVECs treated overnight with TNFα, with or without isolated neutrophils co-incubated for 5 min. Nuclei (blue) and ICAM-1 (green) were co-stained. The area between the dotted orange lines is shown as a Y-Z projection. Orange arrows in the Y-Z projection point to apical filopodia. Zooms of the yellow-outlined region are shown. Scale bar, 20 μm. (E) Bar graph displaying filopodia/μm^2^ + SD. Each dot represents 1 image, and each color represents 1 of 3 biological replicates. Unpaired t test was performed on the medians of each experiment, p = 0.0292. (F) Schematic representation of the neutrophil flow assay with two neutrophil waves. Endothelial cells, treated overnight with TNFα, were subjected to flow for 2 min before neutrophils with a green membrane label were perfused over the endothelial monolayer. After 5 min, a second wave of neutrophils with a far-red membrane label was perfused over the same endothelial cells. After another 15 min, tile-scans were made to measure adhesion and TEM efficacy. (G) Stills at time point from time-lapse from a neutrophil flow experiment, in which neutrophils from the first wave were tracked. Green dots show neutrophils above the endothelial monolayer that are firmly adhered. Magenta dots show neutrophils that have undergone diapedesis. Time starts at first detected adhesion event. Scale bar, 100 μm. (H) Line graph of number of neutrophils above and below the endothelial monolayer at each time point. Indicated are the moment of addition of the first wave (0:00) and the moment the second wave would be added in the 2-wave set-up (5:00). (I) Tile-scan image of an endothelial monolayer at the time of measurement, containing neutrophils of the first (green) and second (magenta) waves. Zooms of the yellow-outlined region are shown below. Scale bar, 500 μm. (J) Violin plot showing the distance of neutrophils of the second wave/random dots to a nearest neutrophil of the first wave. Medians and quartiles are displayed, and the three dots show the medians of each replicate. A total of 7145 neutrophils, or random dots, were measured. Paired t test was performed on the medians, p = 0.0028. (K) Bar graph displaying adhered neutrophils, normalized to the first wave. Each dot corresponds to 1 tile-scan and also 1 biological replicate. SD is shown. Unpaired t test was performed on the raw data, p = 0.0253. (L) Bar graph displaying diapedesis efficacy, normalized to the first wave. SD is shown. Each dot corresponds to 1 tile-scan and also 1 biological replicate. Unpaired t test was performed, p = 0.1822.

Earlier research from us and others demonstrated that neutrophils use hotspots at the endothelial surface to efficiently transmigrate, both *in vivo* and *in vitro*.^6^^,^^8^ In these studies, ICAM-1 was shown to be important in the regulation of these so-called TEM hotspots. Additionally, ICAM-1 is known to localize to apical filopodia, where its adhesive function aids to capture circulating leukocytes.^12^ To capture the dynamics of these apical filopodia during TEM, we used lattice light sheet microscopy (LLSM), allowing high-resolution imaging of living samples in 3 dimensions while minimizing phototoxicity.^26^ By creating a mosaic monolayer of human umbilical vein endothelial cells (HUVECs), expressing different colors of the membrane tag CaaX and introducing neutrophils in a third color, we were able to image membrane dynamics during transmigration events in real time. We found the endothelial membrane to be extremely dynamic, in accordance with our previous research (Videos S1 and S2).^7^ During transmigration of neutrophils, apical endothelial membrane protrusions showed a continuously dynamic behavior (Figure 1A). Previously, we found that neutrophils identify such areas of high membrane dynamics as TEM hotspots.^7^ Interestingly, the dynamics of these membranes increased after neutrophils had crossed. Moreover, in many cases, we found a second neutrophil adhering to and transmigrating near the same endothelial cell within 5 min of the first neutrophil transmigration event. To provide evidence that these membrane protrusions are indeed filopodia, we co-transduced TNFα-inflamed HUVECs with ICAM-1 and LifeAct. Here, we observed that these apical membrane protrusions were highly dynamic and rich for both ICAM-1 and F-actin (Figure 1B and Videos S3, S4, and S5). Scanning electron microscopy also demonstrated that inflamed HUVECs indeed contain apically protruding membrane structures (Figure 1C), even though fixation did cause many of them to collapse.

Video S1. Light-sheet microscopy of neutrophil transmigration, related to Figure 1A Neutrophils labeled with DiD (magenta) transmigrating at a junction of two overnight TNFα-treated CaaX-transduced HUVECs (green and cyan). Scale bar, 5 μm. Total time frame is 560 s.

Video S2. Light-sheet microscopy of neutrophil transmigration from different angle, related to Figure 1A Neutrophils labeled with DiD (magenta) transmigrating at a junction of two overnight TNFα-treated CaaX-transduced HUVECs (green and cyan). Scale bar, 3 μm. Total time frame is 560 s.

Video S3. Light-sheet microscopy of inflamed endothelial cells, related to Figure 1B HUVECs co-transduced with ICAM-1 (green) and F-actin (magenta). Scale bar, 5 μm. Total time frame is 150 s.

Video S4. Light-sheet microscopy of inflamed endothelial cells, related to Figure 1B Same video as S3, but only showing the ICAM-1 (green) channel. Scale bar, 5 μm. Total time frame is 150 s.

Video S5. Light-sheet microscopy of inflamed endothelial cells, related to Figure 1B Same video as S3, but only showing the F-actin (magenta) channel. Scale bar, 5 μm. Total time frame is 150 s.

Based on these findings, we hypothesized that neutrophil adhesion could prime endothelial cells to increase membrane dynamics to allow even more neutrophils to cross. To test this, we added neutrophils to endothelial cells and let them crawl around and transmigrate for 5 min prior to fixation and staining for ICAM-1, known to localize at apical endothelial filopodia (Figure 1D).^12^ We found that endothelial monolayers that experienced neutrophil adhesion showed an increase in the number of filopodia compared to endothelial cells that did not experience any adherent neutrophils (Figure 1E). Y-Z projections of these images showed that these ICAM-1-rich structures indeed protrude from the apical surface (Figure 1D).

To investigate whether the neutrophil-induced increase in filopodia could improve neutrophil adhesion even more, we performed a flow assay in which two subsequent waves of neutrophils, stained in two distinct colors, were perfused over the endothelium with a 5-min interval (Figure 1F). After 5 min, most neutrophils of the first wave had undergone diapedesis, allowing room for neutrophils of the second wave to adhere (Figures 1G and 1H). When observing the location of adhered and transmigrated neutrophils, we found that neutrophils of the second wave were indeed found near neutrophils of the first wave compared to randomly generated spots (Figures 1I and 1J), indicating that neutrophils of both waves underwent TEM at similar regions in the endothelial monolayer. When looking at total neutrophil numbers, neutrophils of the second wave showed an increase in their adhesion up to almost 50% compared to the number of neutrophils from the first wave (Figure 1K). Diapedesis efficacy was not altered between the two waves (Figure 1L). Together, these data suggest that neutrophil adhesion leads to ICAM-1-rich apical filopodia formation in endothelial cells, allowing for more subsequent neutrophil adhesion.

**Figure 2:**
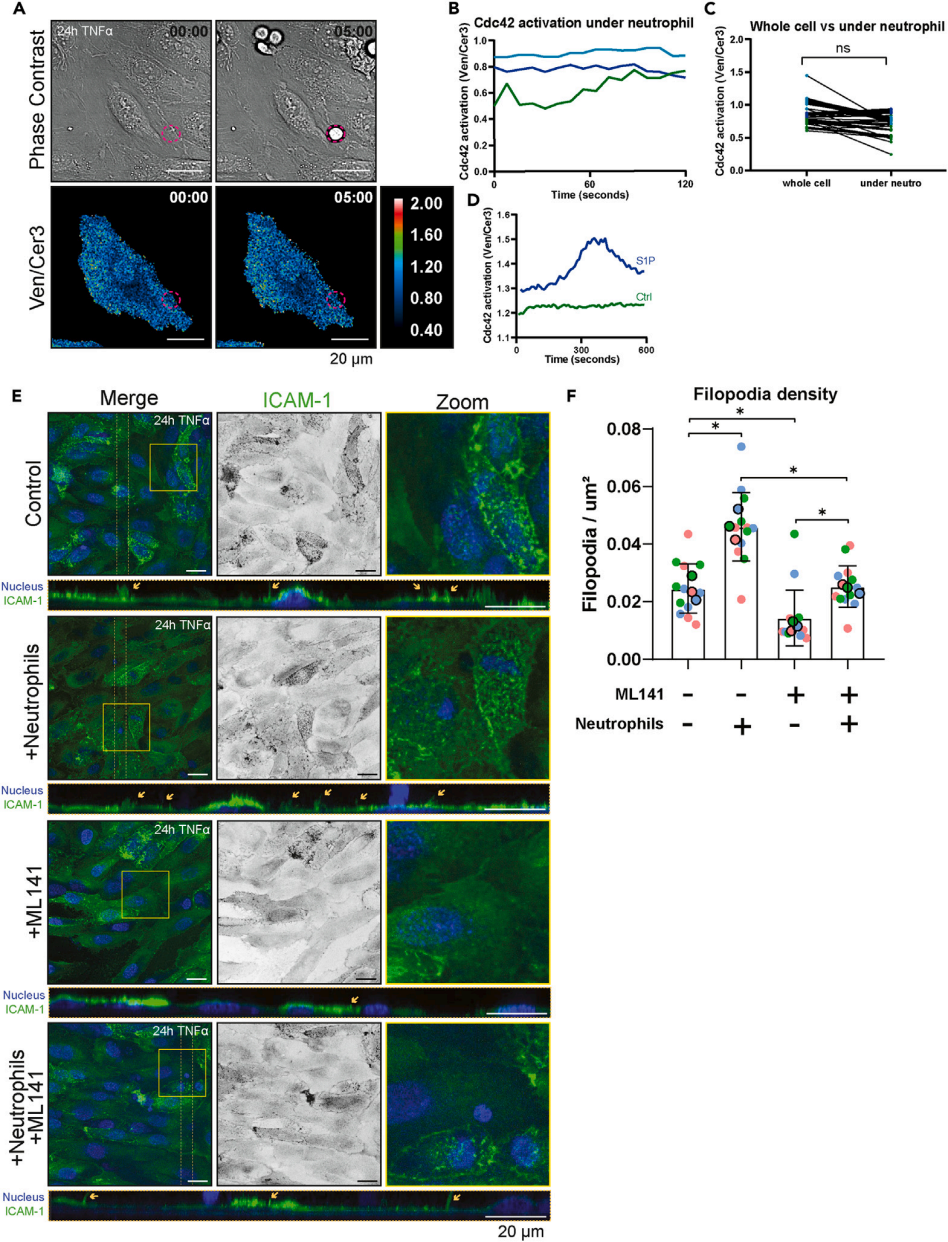
Cdc42 is not activated by neutrophil adhesion (A) Stills from FRET measurements of the Cdc42 sensors during a TEM assay. HUVECs were treated with TNFα overnight. Upper panels show phase-contrast images and bottom images show Venus/Cerulean3 signal of a cell transfected with the Cdc42 FRET sensor, displayed with a pseudo-color LUT. Left panels show prior to neutrophil adhesion, right panels show during neutrophil adhesion. The red-dotted circle displays the location of the neutrophil. Time is annotated in min:sec Scale bar, 20 μm. (B) Line graph showing Cdc42 activation over time across entire endothelial cells (Venus/Cerulean3) of three representative cells from 3 biological replicates. (C) Cdc42 activity (Venus/Cerulean3) of the whole cell versus only the area directly under the neutrophil, measured on the same time point. Each dot represents a single crawling neutrophil and colors represent the three biological replicates. A paired t test on the means of each biological replicate was performed, p = 0.3304. (D) Line graph of Cdc42 activity (Venus/Cerulean3) of a whole endothelial cell, treated with DMSO (green) or sphingosine-1-phosphate (indigo). Graph shows only 1 representative cell. (E) Immunofluorescent stains of HUVECs treated overnight with TNFα, with or without isolated neutrophils co-incubated for 5 min, with or without ML141 (25 μM) co-incubation. Nuclei (blue) and ICAM-1 (green) were co-stained. The area between the dotted orange lines is shown as a Y-Z projection. Orange arrows in the Y-Z projection point to apical filopodia. Zooms of the yellow-outlined region are shown. Scale bar, 20 μm. (F) Bar graph displaying filopodia/μm^2^ + SD. Each dot represents 1 image, and each color represents 1 of 3 biological replicates. Unpaired ANOVA was performed on the medians of each experiment. Control vs. +Neutrophils, p = 0.0003. Control vs. + ML141, p = 0.0096. +ML141 vs. +ML141 +Neutrophils, p = 0.0087. +Neutrophils vs. +ML141 +Neutrophils, p = 0.0096. +ML141 vs. +ML141 +Neutrophils, p = 0.0003.

As endothelial apical filopodia were previously shown to be regulated by the small GTPase Cdc42,^12^ we used a fluorescence resonance energy transfer (FRET)-based Cdc42 biosensor expressed in endothelial cells to measure Cdc42 activation in real time during neutrophil crawling.^27^ We did not find an increase in endothelial Cdc42 activation upon neutrophil adhesion or crawling (Figure 2B). When comparing the FRET signal directly under the crawling neutrophil with the FRET signal in the whole cell, also no heightened Cdc42 activity was measured (Figure 2C). At the end of the experiment, we treated the endothelial cells with sphingosine-1-phosphate (S1P), a well-known activator of Cdc42, as a positive control. As expected, S1P increased Cdc42 activity (Figure 2D). To further exclude the role of Cdc42 in neutrophil-induced filopodia formation, we co-incubated neutrophils and Cdc42 inhibitor ML141 on HUVECs for 5 min. We found that even though ML141 decreased filopodia numbers in basal conditions, there was still an observed increase in filopodia numbers upon neutrophil addition (Figures 2E and 2F). Together, these results indicate that neutrophil adhesion induced filopodia in endothelial cells in a Cdc42-independent manner.

### Generation of KTR sensors in an endothelial line

As neutrophil-induced filopodia formation is Cdc42 independent, we focused on alternative signaling pathways that may induce filopodia. Previous research showed ERK, p38, and JNK activation after ICAM-1 cross-linking and observed that short (1 h) JNK inhibition impaired lymphocyte adhesion.^21^ However, the exact spatiotemporal resolution of the mechanism behind MAPK signaling during TEM has not been extensively studied yet. To study kinase signaling on a single-cell level over time, we employed previously described KTR sensors for the three main MAPK classes (ERK, JNK, and p38) and Akt.^25^^,^^28^ The kinase-specific substrate in each KTR results in the translocation from the nucleus to the cytoplasm when kinase activity is increased. (Figure S1A). When combined with a nuclear marker, the cytoplasm/nucleus (C/N) ratio can be measured and used as readout for kinase activity. To be able to stably express KTR sensors together with a nuclear marker in endothelial cells, we generated two blood outgrowth endothelial cell (BOEC) lines. These cell lines can be maintained in culture for several passages without losing any of the typical endothelial cell characteristics.^29^ In these cell lines, the KTRs and nuclear markers are expressed at equal levels as they are originating from the same plasmid and are separated by self-cleaving peptide 2A sequences.^25^ The first BOEC line contains the Akt-KTR-mTurquoise2 and the ERK-KTR-mNeonGreen sensors and an H2A-mScarlet nuclear marker.^25^ The second BOEC line contains mKO-MK2 (a p38 KTR) and a JNK-KTR-mCherry sensor, with a nuclear localization signal (NLS)-iRFP670-NLS sequence as a nuclear marker.^28^ To prevent crosstalk between the fluorescent proteins, we transfected all single-color constructs into BOECs and imaged them in three different fluorescent channels. In the selected settings, crosstalk is shown to be negligible (Figures S1B and S1C).

To show the functionality of our cell lines and determine the dynamic range of the KTR sensors, BOECs were treated with the inflammatory stimulus TNFα, a known short-term activator of the kinases tested. ERK, JNK, and p38 showed an immediate response to the treatment, evidenced by the translocation of the KTRs (Figures S1D, S1F, and S1G, Videos S6 and S7), indicating the dynamic range of the sensors upon TNFα. Akt translocation was minimal, indicating that the dynamic range of the Akt sensor upon TNFα treatment was limited (Figure S1E, Video S6), which was in accordance with earlier work with these sensors in other cell types.^25^ These experiments show that the KTR sensor BOEC lines adequately respond to TNFα and can be used to further investigate the spatiotemporal kinase signaling during TEM.

Video S6. ERK-KTR/Akt-KTRswith TNFα stimulus, related to Figures S1D and S1E Confocal microscopy time-lapse of ERK-KTR and Akt-KTR expressed in BOECs. On the left, a nucleus marker is shown. The middle panel shows the ERK-KTR. The right panel shows the Akt-KTR. Scale bar, 50 μm. Total time frame is 38 min, TNFα is added after 20 min.

Video S7. JNK-KTR/p38-KTRs with TNFα stimulus, related to Figures S1F and S1G Confocal microscopy time-lapse of JNK-KTR and p38-KTR, expressed in BOECs. On the left, a nucleus marker is shown. The middle panel shows the JNK-KTR. The right panel shows the p38-KTR. Scale bar, 50 μm. Total time frame is 38 min, TNFα is added after 20 min.

### Neutrophil adhesion activates JNK signaling

**Figure 3:**
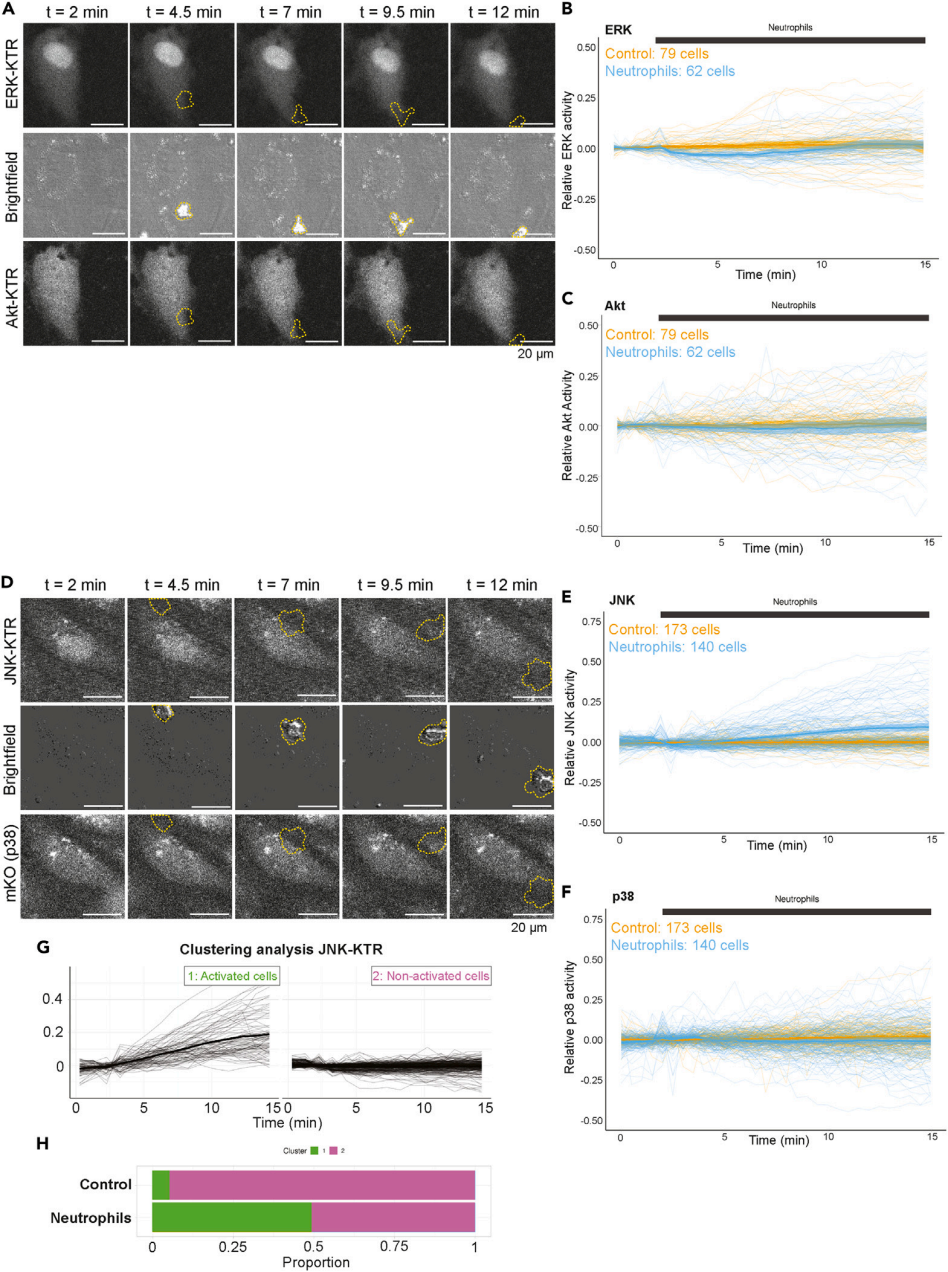
Neutrophil adhesion activates JNK in endothelial cells (A) Stills from a representative cell from the ERK and Akt KTR experiment shown in A and B. The upper panels show the ERK-KTR, the middle panels show a brightfield view and the lower panels show the Akt-KTR. With yellow outlines, the position of the neutrophil is shown. Scale bar, 20 μm. (B) A time-series plot of ERK activation, measured by cytoplasmic/nuclear intensity of the ERK-KTR. (C) A time-series plot of Akt activation, measured by cytoplasmic/nuclear intensity of the Akt-KTR. (B-C) BOECs were treated with TNFα overnight and starved 4 h before imaging. Control cells (orange, 79 cells) and cells with neutrophils co-incubated (blue, 62 cells) are displayed together. Neutrophils were added to the endothelial cells after 2.5 min. The dark thicker line displays the mean of each condition, together with a 95% confidence interval. Data originate from 3 biological replicates. (D) Stills from a representative cell from the JNK and p38 KTR experiment shown in A and B. The upper panels show the JNK-KTR, the middle panels show a brightfield view and the lower panels show the p38-KTR. With yellow outlines, the position of the neutrophil is shown. Scale bar, 20 μm. (E) A time-series plot of JNK activation, measured by cytoplasmic/nuclear intensity of the JNK-KTR. (F) A time-series plot of p38 activation, measured by cytoplasmic/nuclear intensity of the p38-KTR. (E-F) BOECs were treated with TNFα overnight and starved 4 h before imaging. Control cells (orange, 173 cells) and cells with neutrophils co-incubated (blue, 140 cells) are displayed together. Neutrophils were added to the endothelial cells after 2.5 min. The darker and thicker line displays the mean of each condition, together with a 95% confidence interval. Data originate from 3 biological replicates. (G) Time-series plot of the JNK-KTR data after clustering analysis. Data from control and neutrophil conditions were combined and clustered based on Manhattan distance, using a Ward.D2 linkage method, based on two clusters: activated (green) and non-activated (magenta) cells. X axis data are binned in pairs of 2 to smooth out the data, and all timepoints before neutrophil addition were excluded from the clustering algorithm. (H) Results from the clustering analysis, displaying the proportion of endothelial cells that belongs to the activated (green), or non-activated (magenta) clusters in both the control and the neutrophil conditions.

To investigate the role of kinase signaling during TEM of neutrophils, neutrophils were perfused over endothelial cell monolayers expressing one of the two KTR cell lines, and those were overnight treated with TNFα. Next, neutrophils were allowed to adhere and transmigrate through the endothelial monolayer. An excess number of neutrophils were added to make sure that on average each endothelial cell encountered at least one adhering neutrophil. We found that the presence of neutrophils did not change the activity in Akt and p38 (Figures 3A, 3C, 3D, and 3F, Videos S8 and S9). ERK activity decreased slightly as neutrophils adhered to the endothelial monolayer (Figures 3A and 3B, Video S8). However, JNK activity was increased upon neutrophil adhesion to the endothelial monolayer (Figures 3D and 3E, Video S9). Due to the highly dynamic nature of the TEM process, combined with the slight delay in response after a stimulus in the KTR sensors, it is not straightforward to pinpoint exactly which neutrophil TEM event causes JNK activation in which EC. Furthermore, one EC often encountered multiple neutrophils over the course of each experiment, complicating the analysis more. However, on average a clear JNK response was observed (Figures 3D and 3E). These data suggest that neutrophil adhesion increased JNK activity whereas a minimal downstream activity of ERK, and no change in activity for Akt and p38 was detected.

Video S8. ERK-KTR/Akt-KTRs during neutrophil TEM, related to Figure 3A Confocal microscopy time-lapse of neutrophil TEM through ERK-KTR and Akt-KTR expressing BOECs. On the left, the ERK-KTR is shown. The middle panel shows the widefield channel, in which the crawling neutrophil is visible. The right panel shows the Akt-KTR. Scale bar, 50 μm. Scale bar, 20 μm. Total time frame is 15 min, neutrophils are added after 2 min.

Video S9. JNK-KTR/p38-KTRs during neutrophil TEM, related to Figure 3D Confocal microscopy time-lapse of neutrophil TEM JNK-KTR and p38-KTR expressing BOECs. On the left, the JNK-KTR is shown. The middle panel shows the widefield channel, in which the crawling neutrophil is visible. The right panel shows the p38-KTR. Scale bar, 20 μm. Total time frame is 15 min, neutrophils are added after 2 min.

To investigate this further, we combined the datasets for control, i.e., ECs that did not encounter any neutrophils, and ECs that showed neutrophil-induced JNK-KTR response and performed an unbiased clustering analysis to divide all cells into two groups: non-responding and responding ECs (Figure 3G). After a clustering analysis, we show that in the control condition, around 5% of the endothelial cells showed elevated levels of JNK activity, whereas the percentage of responding endothelial cells increased to 50% in the condition where endothelial cells did encounter neutrophils (Figure 3H). In conclusion, we show that neutrophil adhesion rapidly activates JNK in ECs, whereas ERK, Akt, or p38 did not respond. Also, the single-cell data analysis showed that on average 50% of ECs that encounter neutrophils showed increased JNK activity, indicating that not all endothelial cells respond in the same way on neutrophil adhesion.

### JNK1 signaling is required for neutrophil adhesion

**Figure 4:**
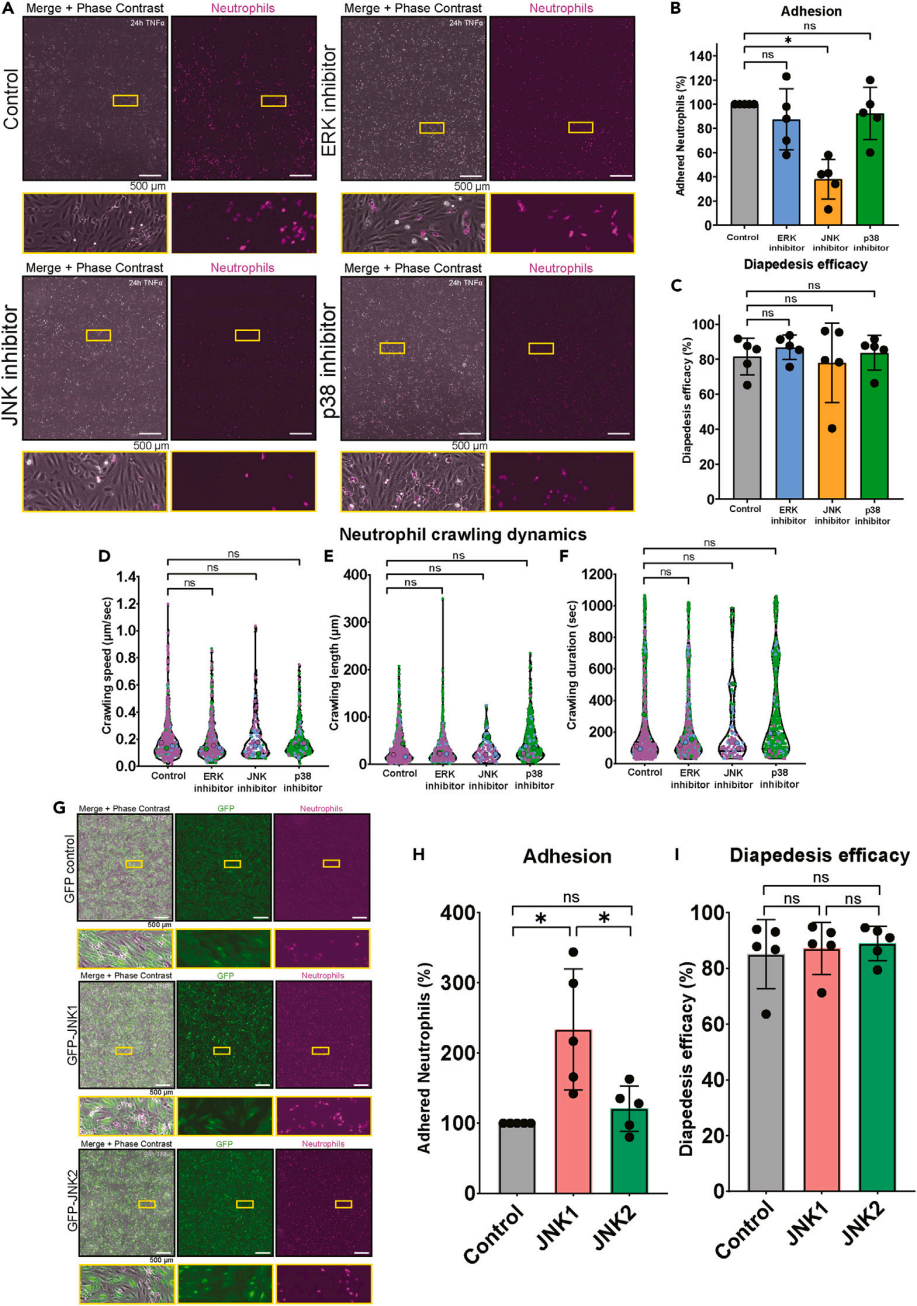
JNK signaling is required for neutrophil adhesion (A) Stills from time-lapse videos from TEM under flow assays. Overnight TNFα-treated HUVECs were treated with MAPK inhibitors for 30 min. Neutrophils were perfused over HUVECs and imaged for 15 min. Here, the state of the experiment at 15 min is shown for each condition. Scale bar, 100 μm. (B) Bar graph displaying adhered neutrophils to overnight TNFα-treated HUVECs treated with MAPK inhibitors for 30 min, normalized to mock-treated HUVECs. SD is shown. Each dot corresponds to 1 tile-scan and also 1 biological replicate. A Repeated-Measure one-way ANOVA was performed on the non-normalized data, comparing all conditions to the control condition. Control vs. ERK inhibitor, p = 0.5947. Control vs. JNK inhibitor, p = 0.0025. Control vs. p38 inhibitor, p = 0.7827. (C) Bar graph displaying efficacy of diapedesis of neutrophils through overnight TNFα-treated HUVECs treated with MAPK inhibitors for 30 min, normalized to mock-treated HUVECs. SD is shown. Each dot corresponds to 1 tile-scan and also 1 biological replicate. A Repeated-Measure one-way ANOVA was performed, comparing all conditions to the control condition. Control vs. ERK inhibitor, p = 0.2114. Control vs. JNK inhibitor, p = 0.8998. Control vs. p38 inhibitor, p = 0.8511. (D) Violin plot displaying crawling speed in μm/s of neutrophils on overnight TNFα-treated HUVECs treated with MAPK inhibitors for 30 min. Each dot corresponds to 1 neutrophil and each color corresponds to 1 biological replicate and also 1 biological replicate. A Repeated-Measure one-way ANOVA was performed on the medians of each biological replicate, which is shown in the bigger black-outlined dot, comparing all conditions to the control condition. Control vs. ERK inhibitor, p = 0.6008. Control vs. JNK inhibitor, p = 0.7282. Control vs. p38 inhibitor, p = 0.7441. (E) Violin plot displaying crawling length in μm of neutrophils on overnight TNFα-treated HUVECs treated with MAPK inhibitors for 30 min. Each dot corresponds to 1 neutrophil and each color corresponds to 1 biological replicate and also 1 biological replicate. A Repeated-Measure one-way ANOVA was performed on the medians of each biological replicate, which is shown in the bigger black-outlined dot, comparing all conditions to the control condition. Control vs. ERK inhibitor, p = 0.9371. Control vs. JNK inhibitor, p = 0.8038. Control vs. p38 inhibitor, p = 0.8560. (F) Violin plot displaying crawling duration in sec of neutrophils on overnight TNFα-treated HUVECs treated with MAPK inhibitors for 30 min. Each dot corresponds to 1 neutrophil and each color corresponds to 1 biological replicate and also 1 biological replicate. A Repeated-Measure one-way ANOVA was performed on the medians of each biological replicate, which is shown in the bigger black-outlined dot, comparing all conditions to the control condition. Control vs. ERK inhibitor, p = 0.9501. Control vs. JNK inhibitor, p = 0.7131. Control vs. p38 inhibitor, p = 0.9941. (G) Stills from time-lapse videos from TEM under flow assays. Overnight TNFα-treated HUVECs were overexpressed GFP-labeled JNK isoforms. Neutrophils were perfused over HUVECs and imaged for 15 min. Here, the state of the experiment at 15 min is shown for each condition. Scale bar, 100 μm. (H) Bar graph displaying adhered neutrophils to overnight TNFα-treated HUVECs overexpressing control GFP, GFP-JNK1 or GFP-JNK2, normalized to control GFP overexpressing HUVECs. Each dot corresponds to 1 tile-scan and also 1 biological replicate. SD is shown. A Repeated-Measure one-way ANOVA was performed on the non-normalized data, comparing all conditions. Control vs. JNK1, p = 0.0034. Control vs. JNK2, p = 0.7444. JNK1 vs. JNK2, p = 0.0090. (I) Bar graph displaying efficacy of diapedesis of neutrophils through overnight TNFα-treated HUVECs overexpressing control GFP, GFP-JNK1 or GFP-JNK2, normalized to control GFP overexpressing HUVECs. Each dot corresponds to 1 tile-scan and also 1 biological replicate. SD is shown. A Repeated-Measure one-way ANOVA was performed on the non-normalized data, comparing all conditions. Control vs. JNK1, p = 0.7674. Control vs. JNK2, p = 0.4447. JNK1 vs. JNK2, p = 0.8375.

To further study the role of kinase signaling during TEM, we employed 30-min chemical inhibition against ERK, p38, and JNK during transmigration-under-flow experiments using primary HUVECs to study in real time the different steps in the TEM cascade: adhesion, crawling, and diapedesis. The results showed that inhibiting ERK and p38 did not decrease the number of neutrophils that adhered to the endothelial monolayer (Figures 4A and 4B, Videos S10, S11, and S13). Inhibition of JNK however significantly hampered neutrophil adhesion (Figures 4A and 4B, Video S12). None of the inhibitors influenced transmigration efficacy (Figures 4A and 4C, Videos S10, S11, and S13). Neutrophil crawling speed, duration, and length of migration were not altered (Figures 4D–4F, Videos S10, S11, and S13). Conversely, when JNK1 was overexpressed in ECs, the total number of adhered neutrophils increased (Figures 4G and 4H, Videos S14 and S15). Overexpression of another isoform of JNK, JNK2, did not result in increased adhesion (Figures 4G and 4H, Videos S14 and S16). In line with the inhibition studies, TEM efficacy was not altered when JNK1 or JNK2 were overexpressed (Figures 4G and 4I, Videos S14, S15, S16, and S17). In conclusion, our data show that endogenous endothelial JNK1 is required for efficient neutrophil adhesion to the endothelium.

Video S17. Leukocyte transendothelial migration under flow with TNF-alpha-treated endothelial cells overexpressing JNK1

Video S10 Neutrophil flow experiments without inhibitor, related to Figures 4A–4F Widefield time-lapses of neutrophil flow experiments. Neutrophils were flown over HUVECs, with 30 min mock treatment. Scale bar, 100 μm. Total time frame is 15 min, neutrophils are added at start of the video.

Video S11. Neutrophil flow experiments with ERK inhibitor, related to Figures 4A–4F Widefield time-lapses of neutrophil flow experiments. Neutrophils were flown over HUVECs, with 30 min ERK inhibitor. Scale bar, 100 μm. Total time frame is 15 min, neutrophils are added at start of the video.

Video S12. Neutrophil flow experiments with JNK inhibitor, related to Figures 4A–4F Widefield time-lapses of neutrophil flow experiments. Neutrophils were flown over HUVECs, with 30 min JNK inhibitor. Scale bar, 100 μm. Total time frame is 15 min, neutrophils are added at start of the video.

Video S13. Neutrophil flow experiments with p38 inhibitor, related to Figures 4A-F Widefield time-lapses of neutrophil flow experiments. Neutrophils were flown over HUVECs, with 30 min p38 inhibitor. Scale bar, 100 μm. Total time frame is 15 min, neutrophils are added at start of the video.

Video S14. Neutrophil flow experiment with GFP overexpression, related to Figures 4G–4I Widefield time-lapses of neutrophil flow experiments. Neutrophils were flown over HUVECs, transduced with GFP control. Scale bar, 100 μm. Total time frame is 15 min, neutrophils are added at start of the video.

Video S15. Neutrophil flow experiment with GFP-JNK1 overexpression, related to Figures 4G–4I Widefield time-lapses of neutrophil flow experiments. Neutrophils were flown over HUVECs, transduced with GFP-JNK1. Scale bar, 100 μm. Total time frame is 15 min, neutrophils are added at start of the video.

Video S16. Neutrophil flow experiment with GFP-JNK2 overexpression, related to Figures 4G–4I Widefield time-lapses of neutrophil flow experiments. Neutrophils were flown over HUVECs, transduced with GFP-JNK2. Scale bar, 100 μm. Total time frame is 15 min, neutrophils are added at start of the video.

### Filopodia formation is regulated by JNK1 signaling

**Figure 5:**
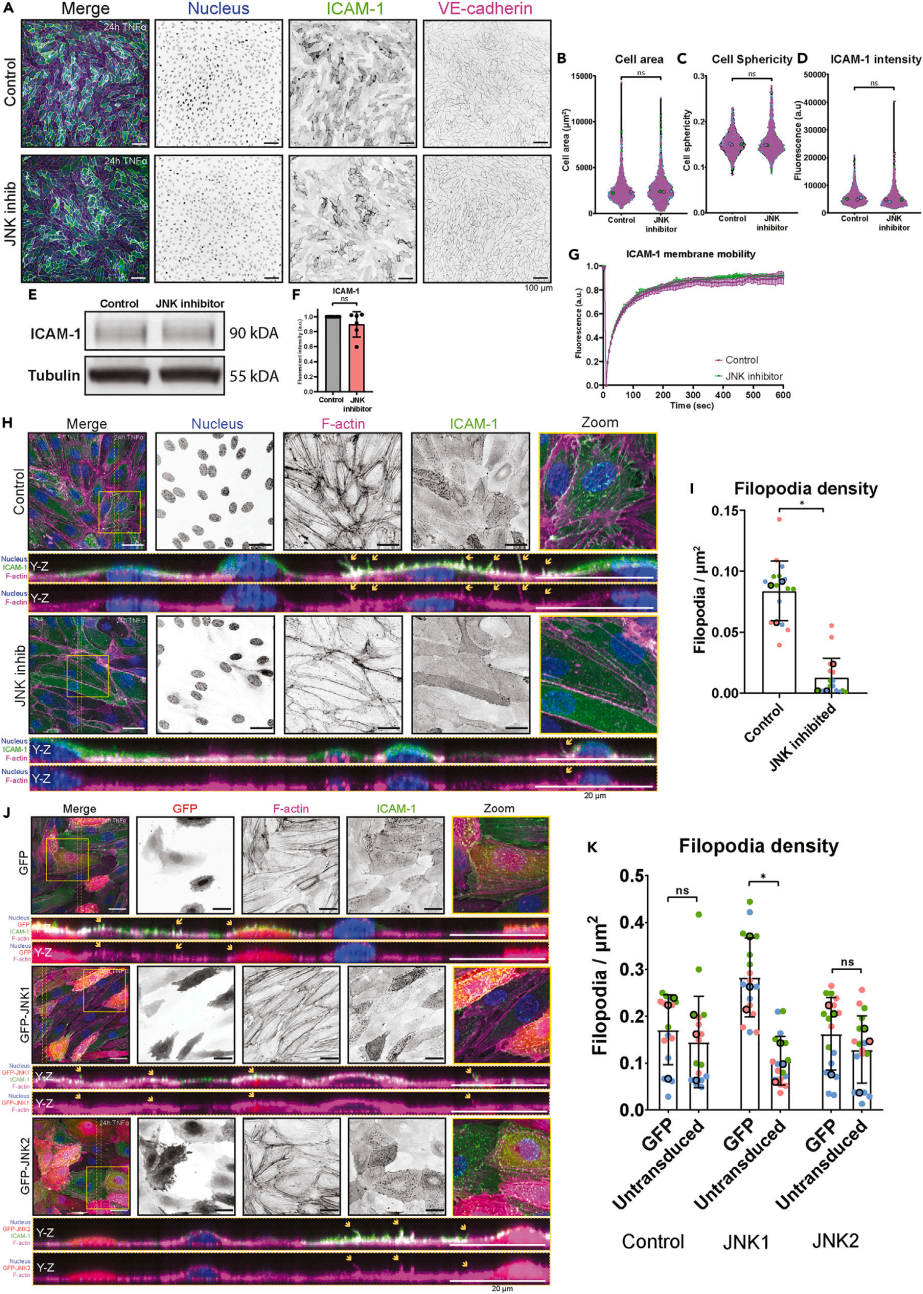
JNK signaling is important for filopodia formation (A) Immunofluorescent images of overnight TNFα-treated HUVECs, treated with or without 30-min JNK inhibitor. HUVECs were stained for nuclei (blue), ICAM-1 (green) and VE-cadherin (magenta). Scale bar, 100 μm. (B) Violin plot displaying cell area in μm^2^ of control and JNK-inhibited HUVECs, which were treated overnight with TNFα. Each dot corresponds to 1 HUVEC and each color corresponds to 1 biological replicate. Unpaired t test was performed on the medians, displayed with larger black-outlined dots, of each replicate, p = 0.1509. (C) Violin plot displaying cell sphericity of control and JNK-inhibited HUVECs, which were treated overnight with TNFα. Each dot corresponds to 1 HUVEC and each color corresponds to 1 biological replicate. Unpaired t test was performed on the medians, displayed with larger black-outlined dots, of each replicate, p = 0.1312. (D) Violin plot displaying ICAM-1 fluorescent intensity of control and JNK-inhibited HUVECs, which were treated overnight with TNFα. Each dot corresponds to 1 HUVEC and each color corresponds to 1 biological replicate. Unpaired t test was performed on the medians, displayed with larger black-outlined dots, of each replicate, p = 0.1435. (E) Western blot of control and JNK-inhibited HUVECs, stained for ICAM-1 and tubulin. (F) Bar graph displaying the quantification of the western blot for ICAM-1. Unpaired t test was performed, p = 0.1746. (G) Time-series graph displaying fluorescent intensity after photobleaching of ICAM-1-GFP in HUVECs treated overnight with TNFα. The mean time-series of control (magenta, 10 cells) and JNK-inhibited (green, 10 cells) HUVECs are shown, together with a 95% confidence interval. Data originates from 3 biological replicates. Each cell time-series is normalized to its intensity at 0 s (100%) and 15 s (0%). (H) Immunofluorescent stains of HUVECs treated overnight with TNFα, with or without 30-min JNK-inhibitor treatment. Nuclei (blue), F-actin (magenta) and ICAM-1 (green) were co-stained. The area between the dotted orange lines is shown as a Y-Z projection. Orange arrows in the Y-Z projection point to apical filopodia. Zooms of the yellow-outlined region are shown. Scale bar, 20 μm. (I) Bar graph displaying filopodia/μm^2^ of HUVECs treated overnight with TNFα, with or without 30-min JNK inhibition. Each dot represents 1 image, and each color represents 1 of 3 biological replicates. Unpaired t test was performed on the medians of each experiment, p = 0.0057. (J) Immunofluorescent stains of HUVECs treated overnight with TNFα, overexpressing either control GFP (upper panels), GFP-JNK1 (middle panels), or GFP-JNK2 (lower panels) in a mosaic manner. Nuclei (blue), F-actin (magenta) and ICAM-1 (green) were co-stained. GFP constructs are shown in red. The area between the dotted orange lines is shown as a Y-Z projection. Orange arrows in the Y-Z projection point to apical filopodia. Zooms of the yellow-outlined region are shown. Scale bar, 20 μm. (K) Bar graph displaying filopodia/μm^2^ of HUVECs overexpressing either control GFP (upper panels), GFP-JNK1 (middle panels), or GFP-JNK2 (lower panels) in a mosaic manner. Cells overexpressing the GFP construct were compared with untransduced cells in the same image. Each dot represents 1 image, and each color represents 1 of 3 biological replicates. two-Way ANOVA was performed on the medians of each replicate, comparing overexpressing versus untransduced cells for each construct was performed. Control, p = 0.3875. JNK1, p = 0.0020. JNK2, p = 0.1752.

To investigate why JNK signaling is required for neutrophil adhesion to the endothelium, we focused on endothelial cell morphology upon the inhibition of JNK. Using VE-cadherin as a cell-cell junction marker, we segmented individual ECs within an EC monolayer (Figure 5A). Using this analysis, we did not measure any differences in endothelial cell area (Figure 5B) and sphericity (Figure 5C). As we found reduced neutrophil adhesion in JNK-inhibited endothelial cells, we focused on endothelial ICAM-1, a well-known endothelial regulator of leukocyte adhesion.^30^ However, ICAM-1 surface expression levels based on fluorescent intensity of microscopy images were not altered because of JNK inhibition (Figure 5D), nor was ICAM-1 total protein level (Figures 5E and 5F). Additionally, ICAM-1 membrane motility, determined by fluorescence recovery after photobleaching (FRAP), was also unaltered upon JNK inhibition (Figure 5G).

When we imaged at higher resolution, we observed a change in the actin cytoskeleton structure upon JNK inhibition (Figure 5H). We noticed reduced actin stress fibers and a large reduction in ICAM-1-containing apical filopodia (Figures 5H and 5I). X-Y projections of these images showed that the apical ICAM-1-rich filopodia were also positive for F-actin (Figure 5H). By generating a mosaic endothelial monolayer, in which half of ECs were not transduced and served as control, and the other half was transduced with either a control GFP, GFP-JNK1, or GFP-JNK2, we found that JNK1 increased filopodia density, whereas JNK2 or GFP did not (Figures 5J and 5K). These results indicated that JNK1 is involved in regulating endothelial filopodia formation.

### MARCKSL1 is responsible for filopodia formation

**Figure 6:**
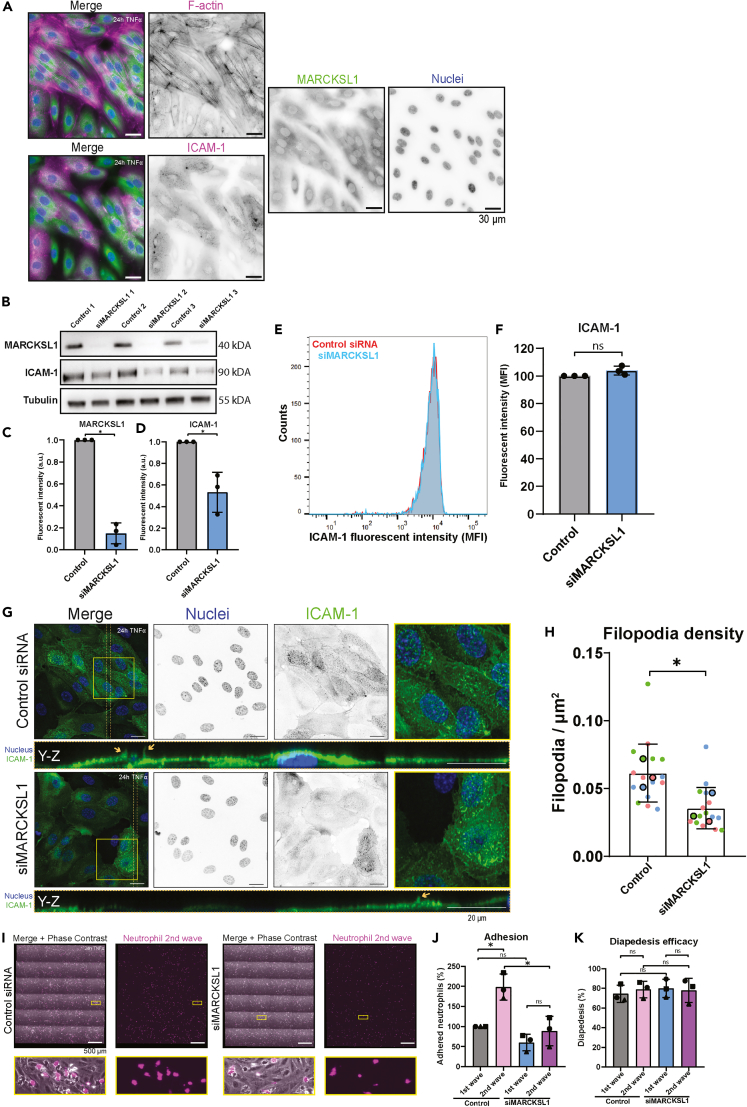
MARCKSL1 regulates neutrophil-induced filopodia (A) Immunofluorescent images of overnight TNFα-treated HUVECs. HUVECs were co-stained for nuclei (blue), ICAM-1 (magenta, lower panel), F-actin (magenta, upper panel) and MARCKSL1 (green). Scale bar, 30 μm. (B) Western blot of HUVECs treated overnight with TNFα, transfected with control or MARCKSL1 siRNA. Blots were stained for MARCKSL1, ICAM-1 and Tubulin. (C) Quantification of the MARCKSL1 fluorescent intensity shown in figure B. Unpaired t test was performed, p < 0.0001. (D) Quantification of the ICAM-1 fluorescent intensity shown in figure B. Unpaired t test was performed, p = 0.0121. (E) Histogram displaying ICAM-1 fluorescent intensity measured with flow cytometry in control and siMARCKSL1 HUVECs, treated 24 h with TNFα. (F) Bar graph displaying quantification of the flow cytometry experiment in Figure E. Unpaired t test was performed, p = 0.1063. (G) Immunofluorescent images of overnight TNFα-treated HUVECs, with control siRNA in the upper panels or siMARCKSL1 in the lower panels. HUVECs were co-stained for nuclei (blue) and ICAM-1 (green). The area between the dotted orange lines is shown as a Y-Z projection. Orange arrows in the Y-Z projection point to apical filopodia. Scale bar, 20 μm. (H) Bar graph displaying filopodia/μm^2^ + SD in HUVECs treated overnight with TNFα, with control siRNA or siMARCKSL1. Each dot represents 1 image, and each color represents 1 of 3 biological replicates. Unpaired t test was performed on the medians of each experiment, p = 0.0423. (I) Tile-scan image of an endothelial monolayer, treated overnight with TNFα, at the time of measurement, containing neutrophils of the first (green) and second (magenta) waves. Upper panels were treated with Control siRNA and lower panels were treated with siMARCKSL1. Zooms of the yellow-outlined region are shown below. Scale bar, 500 μm. (J) Bar graph displaying adhered neutrophils of a first and second neutrophil wave with 5 min in between to overnight TNFα-treated HUVECs treated with control siRNA or siMARCKSL1. Each dot corresponds to 1 tile-scan and also 1 biological replicate. SD is shown. One-way ANOVA was performed on the raw data, comparing all conditions. Control 1^st^ wave vs. Control 2^nd^ wave, p = 0.0084. Control 1^st^ wave vs. siMARCKSL1 1^st^ wave, p = 0.3292. Control 1^st^ wave vs. siMARCKSL1 2^nd^ wave, p = 0.9529. Control 2^nd^ wave vs. siMARCLKSL1 1^st^ wave, p = 0.0010. Control 2^nd^ wave vs. siMARCKSL1 2^nd^ wave, p = 0.0044. siMARCKSL1 1^st^ wave vs. siMARCKSL1 2^nd^ wave, p = 0.5825. (K) Bar graph displaying efficacy of diapedesis of neutrophils of a first and second neutrophil wave with 5 min in between through overnight TNFα-treated HUVECs treated with control siRNA or siMARCKSL1. Each dot corresponds to 1 tile-scan and also 1 biological replicate. SD is shown. One-way ANOVA was performed on the raw data, comparing all conditions. Control 1^st^ wave vs. Control 2^nd^ wave, p = 0.9436. Control 1^st^ wave vs. siMARCKSL1 1^st^ wave, p = 0.8963. Control 1^st^ wave vs. siMARCKSL1 2^nd^ wave, p = 0.9720. Control 2^nd^ wave vs. siMARCLKSL1 1^st^ wave, p = 0.9989. Control 2^nd^ wave vs. siMARCKSL1 2^nd^ wave, p = 0.9993. siMARCKSL1 1^st^ wave vs. siMARCKSL1 2^nd^ wave, p = 0.9928.

To investigate the molecular mechanism by which JNK1 mediates filopodia formation, we focused on MARCKSL1 as this protein is known to be directly phosphorylated by JNK and known as actin-interacting protein.^20^^,^^31^^,^^32^ Using immunofluorescence, we found MARCKSL1 to localize in the cytosol, with no particular colocalization to F-actin or ICAM-1 (Figure 6A). To investigate whether MARCKSL1 plays a functional role in apical filopodia formation, we used a small interfering RNA (siRNA)-based knockdown approach to reduce MARCKSL1 levels (Figures 6B and 6C). This siRNA-based approach did reduce total ICAM-1 protein levels (Figures 6B and 6D), but ICAM-1 surface levels were unaltered (Figures 6E and 6F). Importantly, filopodia density in TNFα-treated ECs was significantly reduced upon silencing of MARCKSL1 (Figures 6G and 6H). Functionally, silencing MARCKSL1 in TNFα-treated ECs resulted in a small non-significant decrease in neutrophil adhesion (Figures 6I and 6J), whereas diapedesis efficacy was unaltered (Figures 6I and 6J). Interestingly, depletion of MARCKSL1 prevented the increase of secondary neutrophil adhesion of a neutrophil wave administrated 5 min after a first wave to the endothelium (Figure 6J), whereas diapedesis efficacy was unaltered, in line with the JNK data (Figure 6K). These data demonstrate that in endothelial cells, MARCKSL1 is involved in apical filopodia formation. Overall, we postulate that primary neutrophil adhesion locally triggers the activity of JNK1, resulting in a local increase in filopodia and, as a functional consequence, an increase in secondary neutrophil adhesion. These data put forward the importance of endothelial TEM hotspots to optimally regulate the maintenance of endothelial integrity during leukocyte TEM under inflammatory conditions.

## Discussion

Adhesion of neutrophils to the inflamed endothelium triggers a variety of intracellular signaling pathways, regulating cell-cell junction remodeling, membrane dynamics, and chemokine secretion, all required to efficiently cross the vascular endothelial barrier.^1^^,^^2^^,^^5^^,^^33^ However, most studies, including ours, that have focused on signaling pathways used biochemical approaches to study intracellular signaling during this very fast and dynamic process. Moreover, in many studies, signaling is typically induced by antibody-coated beads or cross-linking antibodies, to avoid any crosstalk between two cell types present in downstream assays.^21^^,^^34^^,^^35^^,^^36^^,^^37^ Although very valuable, these studies lack spatial and temporal information and the presence of immune cells. Here, we employed advanced imaging techniques to study both membrane dynamics during neutrophil transmigration and the pathways activated downstream of neutrophil adhesion in high spatiotemporal resolution via translocation-based biosensors to elucidate why neutrophils prefer to follow each other during TEM.

We observed that neutrophil adhesion triggered the formation of filopodia and found no activation of the small GTPase Cdc42, but instead increased activation of the MAP kinase JNK, but not ERK, Akt, and p38. In addition, the actin-binding protein MARCKSL1 was found to regulate filopodia formation and secondary neutrophil adhesion. Based on our data, we suggest that initial neutrophil adhesion locally triggers JNK1 to subsequently bind to its downstream substrate MARCKSL1 that then induces filopodia, resulting in more neutrophil adhesion. In the end, this functionally results in increased neutrophil transmigration, although the percentage adherent neutrophils that transmigrated did not change. Based on these data, we concluded that the JNK1-MARCKSL1-filopodia axis works on the adhesion step and not on the diapedesis step. However, we do not show direct evidence that JNK1 phosphorylates MARCKSL1 in our ECs, basing this part of our hypothesis on previous research of other groups. Additionally, we cannot exclude that other molecules could be involved upstream of JNK or downstream of MARCKSL1, or even parallel to MARCKSL1. Especially since MARCKSL1 knockdown does not lead to a filopodia reduction as severe as JNK inhibition, perhaps other JNK substrates are also involved in filopodia formation.

We postulate that the neutrophil-induced increase in filopodia allows more neutrophils to adhere and transmigrate. This may partially explain the TEM “hotspot” phenomenon previously described.^6^^,^^8^ Previous research described a mechanism similar to ours, in which already adherent neutrophils were described to provide binding to subsequently arriving neutrophils via L-selectin. However, we argue that this mechanism most likely takes place earlier in the TEM cascade compared to ours as this paper reports that secondary neutrophil adhesion to previously adherent neutrophils occurs within 30–60 s. In our study, we show that increased leukocyte adhesion still happens 5 min after an initial wave, a time point at which 90% of neutrophils from the first wave have already undergone diapedesis. Furthermore, our flow experiments were performed at 0.8 dyne/cm^2^, a shear stress at which the authors barely observed any L-selectin-mediated neutrophil-neutrophil adhesion. However, studying the relation between both reported mechanisms could be an interesting future approach.

The small GTPase Cdc42 is a Rho GTPase classically associated with filopodia formation^38^ and has indeed been associated with the formation of apical filopodia in endothelial cells downstream of TNFα signaling.^12^ In this study, we showed that overnight depletion of Cdc42 resulted in a decrease in filopodia numbers and neutrophil adhesion. Furthermore, we showed a role for JNK in this pathway, being responsible for the expression of MyosinX. However, neutrophil adhesion did not induce local and direct Cdc42 activation in this study. Additionally, Cdc42 inhibition did not prevent neutrophil-induced filopodia formation. We hypothesize that Cdc42- and MARCKSL1-induced filopodia formation pathways work in concert under inflammatory conditions. However, once neutrophils adhere, only the JNK1-MARCKSL1 pathway is responsible for filopodia formation via a quick pathway and not depending on gene expression regulation. Finally, based on our data here, we cannot exclude a role of MyosinX in this short-term filopodia formation.

Strikingly, we show that not all MAPKs are activated upon neutrophil adhesion. These data are in line with earlier data on Akt, which was found not to be activated upon ICAM-1 clustering.^17^ However, ICAM-1 clustering did show ERK and p38 activation.^21^^,^^39^ As ICAM-1 is one of the major adhesion molecules involved in neutrophil adhesion, it was expected for ERK and p38 to also be activated using the KTR setup. One explanation for this discrepancy may be the extracellular trigger. The described studies mimicked leukocyte adhesion by specifically cross-linking ICAM-1 with antibodies or using anti-ICAM-1 antibody-coated beads. Taking our data into account, this may indicate that the rate of ICAM-1 signaling induced by neutrophils is lower than when triggering ICAM-1 signaling through antibody-mediated assays and therefore fail to activate ERK and p38.

Our chemical inhibition experiments showed that inhibition of JNK, but not that of ERK or p38, decreased neutrophil adhesion. These data are in line with earlier work, in which a very similar experiment was performed.^21^ In this study, JNK inhibition resulted in reduced Th1 lymphocyte migration across rat brain endothelial cells. However, a study using HUVECs and neutrophils showed that ERK inhibition was detrimental for neutrophil TEM.^40^ This study suggested that ERK signaling in ECs is not required for adhesion, which is in accordance with our study. They suggest that ERK signaling may be required for post-adhesion steps in the TEM cascade. ERK therefore may be involved in the post-diapedesis step, to release neutrophils from the endothelium or during the subendothelial crawling step. However, we did not follow the neutrophils post-diapedesis.

Three very closely related JNK genes encode for JNK1, JNK2, and JNK3, the former two being ubiquitously expressed and the latter being restricted to the central nervous system.^20^ Each of these very closely related proteins has several splicing isoforms. The different JNK proteins and isoforms each display considerably different enzymatic and catalytic activities, varying between substrates. In neurons, MARCKSL1 has been demonstrated to be phosphorylated by all three JNK isoforms, but most potently by JNK1.^31^ Furthermore, previous research overexpressed dominant negative JNK1 and JNK2 and found that only the modified JNK1 construct hampered T cell TEM.^21^ These data fit with our findings that JNK1 overexpression but not JNK2 overexpression improved neutrophil adhesion. Overexpressing constitutively active JNK1 may be an interesting approach to promote leukocyte adhesion and thereby transmigration for therapeutic treatments where elevated leukocyte recruitment is wanted, like immune cell therapy.

In endothelial cells, MARCKSL1 overexpression was shown to induce linear actin bundles and thus filopodia *in vivo.*^32^ However, this paper did not only report effects on apical actin properties but also showed effects on the basal side of the endothelial monolayer. In our studies, we use 2D *in vitro* approaches, which may be a reason that we did not observe notable effects on the basal side. Further evidence that MARCKSL1 is important for filopodia formation comes from work done in neurons, where MARCKSL1 mutants resulted in both lower filopodia density and reduced filopodia dynamics.^31^

We observed heterogeneity in JNK activation between ECs in their response to neutrophil interactions, whereas TNFα-induced JNK translocation showed a homogeneous response. In our experimental setup, all ECs should have been in contact with a neutrophil, but only 50% of the ECs showed measurable nuclear export of the JNK-KTR. One explanation for the heterogeneity in responsiveness may lie in the heterogeneous expression of many of the endothelial adhesion molecules.^8^ Heterogeneous expression of adhesion receptors could potentially explain differences in downstream signaling output.

Endothelial membrane dynamics have been shown to be markers of neutrophil extravasation sites.^7^ In the previous study, we showed that Rac1-induced apical membrane protrusions at junction regions were preferred by neutrophils to cross, thereby serving as TEM hotspots. To what extent these junction membrane protrusions are related to the apical filopodia described here is not known. It could very well be that both apical membrane protrusions work in concert to allow efficient transmigration of leukocytes with limited losses of vascular integrity.

In summary, we describe a pathway involving JNK1 and MARCKSL1, which is triggered upon neutrophil adhesion and increased filopodia formation in ECs, allowing a second wave of neutrophils to adhere and undergo TEM. These data provide a new concept to target accelerated neutrophil extravasation during disease settings.

### Limitations of study

We have used biosensors to track kinase activity with relatively high temporal resolution on a single-cell level. However, both spatially and temporally there is room for improvement. This type of sensor does not report subcellular localization, and their response time is not as quick as certain other sensors since there is a slight delay because of the required nuclear translocation. Furthermore, we have not shown a direct link between JNK1 and MARCKSL1 here, and based part of our conclusion on previous research performed not in an endothelial model system. Additionally, we cannot exclude that there are other JNK1 substrates that are also important for filopodia regulation.

## STAR★Methods

### Key resources table

**Table:** 

REAGENT or RESOURCE	SOURCE	IDENTIFIER
**Antibodies**		

Mouse anti-ICAM-1 Alexa Fluor 647 antibody	BioRad	MCA1615A647T, RRID:AB_2122038
Mouse anti ICAM-1 Alexa Fluor 546 antibody	Santa Cruz	Cat# sc-107 AF 546, RRID:AB_627120
Mouse anti-VE-cadherin Alexa Fluor 488 antibody	Abcam	Cat# ab272345
Mouse anti-VE-cadherin Alexa Fluor 647 antibody	BD	Cat# 561567, RRID:AB_10712766
Rabbit anti ICAM-1 antibody	Santa Cruz	Cat# sc-7891, RRID:AB_647486
Rabbit anti MARCKSL1 antibody	Abcam	Cat# ab184546, RRID:AB_2858219
Donkey anti-Rabbit Alexa Fluor 555 antibody	Molecular Probes	Cat# A-31572, RRID:AB_162543
Mouse anti alpha-tubulin antibody	Sigma	Cat# T6199, RRID:AB_477583
Goat Anti-mouse HRP antibody	Agilent	Cat# P0447, RRID:AB_2617137
Swine Anti-rabbit HRP antibody	Agilent	Cat# P0399, RRID:AB_2617141

**Chemicals, peptides, and recombinant proteins**		

Hoechst 33342	LifeTechnologies	H-1399
Alexa Fluor 488 Phalloidin	Invitrogen	A12379
Texas Red Phalloidin	Invitrogen	T7471
TransIT	Myrus	MIR-2300
LentiX concentrator	Clontech	631232
Puromycin	Invivogen	Ant-pr-1
PD184352	Sigma	PZ0181
SP600125	Sigma	S5567
SB203580	Sigma	S9307
Accutase	Sigma	A6964
Near-IR Life-dead marker	Invitrogen	L10119A
Hexamethyldisilane	Sigma	217069
Leit-C carbon cement	PLANO	G3300
Milli-Q	Gibco	A1283-01
Human serum albumin	Sanquin Reagents	N/A
VybrantTM DiD Cell-labelling solution	Invitrogen	V22887
VybrantTM DiO Cell-labelling solution	Invitrogen	V22886
TNFα	Peprotech	300-01A
Fibronectin	Sanquin Reagents	N/A
CellTracker Red	Invitrogen	C34552
Opti-MEM	Gibco	31985062

**Critical commercial assays**		

4-12% NuPage Bis-Tris Gel	Invitrogen	NP0322BOX
iBlot2 Gel Transfer device	Invitrogen	IB21001
Nitrocellulose membrane	Invitrogen	IB23001
Pierce ECL Western Blotting Substrate kit	ThermoFisher	32106

**Experimental models: Cell lines**		

HUVEC	Promocell	C-22011
BOEC	This paper	N/A
HEK-293T	ATCC	CRL-3216

**Oligonucleotides**		

See Table S1		

**Recombinant DNA**		

pLV-GFP-JNK1	This Paper	N/A
pLV-GFP-JNK2	This Paper	N/A
pLV-ICAM-1-GFP	This Paper	N/A
pLV-mNeongreen-CaaX	Arts et al.^7^	N/A
pLV-mTurquoise2-CaaX	Arts et al.^7^	N/A
ERK-Akt PiggyBac	Chavez-Abiega et al.^25^	N/A
Transposase	Chavez-Abiega et al.^25^	N/A
JNK-KTR-mCherry	Addgene	plasmid # 115493
mKO-MK2	Addgene	plasmid # 115492
JNK-p38 PiggyBac	Addgene	plasmid # 115494
pNLS-iRFP670	Addgene	plasmid # 45466
mNeonGreen-C1	Joachim Goedhart	N/A
mTurquoise2-C1	Addgene	plasmid # 54842
3x-NLS mScarletI	Addgene	plasmid # 98816

**Software and algorithms**		

FlowJo Version 10 for Windows	Becton, Dickinson and Company	N/A
Imaris Version 10.0	Oxford instruments	RRID:SCR_007370
Fiji version 1.52p	Schindelin et al.^45^	N/A
CellProfiler 3.0.0	Stirling et al.^47^	N/A
Plottwist	Goedhart^48^	N/A

**Other**		

Ibidi μ-slides VI^0.4^	Ibidi	80606-90
5 mm round coverslips	Warner instruments	C5-5R
Tetraspeck beads	Invitrogen	T7280

### Resource availability

#### Lead contact

Further information and requests for resources and reagents should be directed to and will be fulfilled by the lead contact, Jaap D. van Buul (j.d.vanbuul@amsterdamumc.nl).

#### Materials availability

Plasmids and stable cell lines generated in this study are available upon request with the lead contact.

### Experimental model and subject details

#### Cell lines and primary cells

HUVECs were cultured on fibronectin (FN)-coated dishes, using Endothelial Basal Medium, supplemented with singlequots (Promocell, C-22011) and 100 U/mL penicillin and streptomycin (P/S) at 37ᵒC in 5% CO_2_ to create Endothelial Cell Growth Medium 2 (EGM-2). HUVECs were used between passage 4 and 7 and were never grown to grow above 70% confluency until a monolayer was required for an experiment. BOECs were cultured on 0.1% gelatin-coated dishes. For BOECs, 1:5 fetal calf serum (FCS) (Bodinco, Alkmaar, The Netherlands) was added to EGM-2, creating EGM-18 medium. To mimic inflammation in HUVECs or BOECs, TNFα (Peprotech, 300-01A) was added 10 ng/mL overnight to induce inflammatory protein expression in ECs. HEK-293T were purchased from ATCC (CRL-3216) and were cultured in Dulbecco’s Modified Eagle Medium (DMEM) (Gibco, 41965-039) containing 10% fetal calf serum, 100 U/mL P/S. Primary human neutrophils were collected from peripheral blood extracted from healthy voluntary donors, employed at Sanquin in The Netherlands, that signed informed consent according to the rules maintained by the Sanquin Medical Ethical Committee, which are based on rules and legislation in place within The Netherlands.

### Method details

#### Plasmids

GFP-JNK1 (Addgene plasmids # 86830) and GFP-JNK2 were a gift from Roney Seger and (Addgene plasmid # 86831)^18^ From these plasmids, the GP-tagged JNK isoforms were amplified using PCR using primer 1 and 2 as forward and reverse primers on the GFP-JNK1 plasmid and using primer 1 and 3 as forward and reverse primers on the GFP-JNK2 plasmid. Both inserts were cloned into a lentiviral backbone using Gibson cloning that was cut open with AgeI (Table S1), using primers 4 and 5 to create pLV-GFP-JNK1 and using primers 6 and 7 to create pLV-GFP-JNK2 (Table S1). pLV-ICAM-1-GFP was described before.^8^ pLV-mNeongreen-CaaX and pLV-mTurquoise2-CaaX were constructed and described earlier.^7^ The ERK-Akt PiggyBac plasmid and the transposase plasmid were described earlier.^25^ The JNK-p38 PiggyBac plasmid (Addgene plasmid # 115494), mKO-MK2 (Addgene plasmid # 115492), JNK-KTR-mCherry (Addgene plasmid # 115492) were a kind gift from Kazuhiro Aoki.^28^ pNLS-iRFP670 was a kind gift from Vladislav Verkhusha (Addgene plasmid # 45466).^41^ mNeonGreen-C1 was a kind gifts of Joachim Goedhart. mTurquoise2-C1 (Addgene plasmid # 54842) and 3x-NLS mScarletI (Addgene plasmid # 98816) were kind gifts of Dorus Gadella.^42^^,^^43^

#### Virus production in HEK-293T

By transfection of third generation lentiviral packaging plasmids in HEK-293T with TransIT (Myrus, MIR 2300) according to the manufacturers protocol, lentiviral particles containing pLV plasmids were generated. The first day after transfection, medium was refreshed earliest in the morning. Lentivirus-containing supernatant was harvested the second and third day after transfection, filtered (0.45 micron) and concentrated with Lenti-X concentrator (Clontech, 631232). Virus was added to HUVEC or BOECs 1:250 to 1:500, depending on the efficacy of the virus. Two-day puromycin-selection (1.5 μg/mL) (InvivoGen, ant-pr-1) was performed 2 to 3 days after transduction.

#### siRNA knockdowns

MARCKSL1 knockdowns were generated using four pooled siRNAs (Horizon Discovery SMARTpool). The siRNA sequences are shown in Table S1. As a control, negative control siRNA was used (Qiagen, 1022076). Microporation was used to transfect HUVECs with siRNA. For each microporation, 500.000 HUVECs and 100 nM siRNA were mixed. After microporation, 90% of this mixture was seeded into a 6 well plate for western blot analysis of knockdowns and 10% was seeded into Ibidi flow slides for either a flow experiment or an IF stain.

#### Inhibitors

ERK was inhibited with 2 μM PD184352 (Sigma, PZ0181) for 30 min. JNK was inhibited with 25 μM SP600125 (Sigma, S5567) for 30 minutes. Finally, p38 was inhibited using 10 μM SB203580 (Sigma, S8307) for 30 minutes. These concentrations and incubation times were based on previous research from our group.^44^ In control samples, cells were treated with DMSO.

#### Antibodies and immunofluorescent stains

Hoechst 33342 (IF, 1:50.000) was bought from LifeTechnologies (H-1399). Mouse anti-ICAM-1 Alexa Fluor 647 (IF, 1:400; Flow Cytometry, 1:400) was bought from BioRad (MCA1615A647T). Mouse anti ICAM-1 Alexa Fluor 546 (IF, 1:400) was bought from Santa Cruz (sc-107 AF546). Mouse anti-VE-cadherin Alexa Fluor 488 (IF, 1:400) was bought from Abcam (ab272345). Mouse anti-VE-cadherin Alexa Fluor 647 (IF, 1:400) was bought from BD Biosciences (561567). Alexa Fluor 488 Phalloidin (IF, 1:500) was bought from Invitrogen (A12379). Texas Red Phalloidin (IF, 1:500) was bought from Invitrogen (T7471). Rabbit anti ICAM-1 antibody (WB 1:500) was purchased from Santa Cruz (SC-7891). Rabbit anti MARCKSL1 antibody (IF, 1:100; WB, 1:500) was purchased from Abcam (ab184546). Donkey anti-Rabbit Alexa Fluor 555 (IF, 1:200) was purchased from Molecular Probes (A31572). Mouse anti alpha-tubulin antibody (WB, 1:5.000) was bought from Sigma (T6199-200UL). Goat Anti-mouse HRP antibody was purchased from Agilent (P0447). Swine Anti-rabbit HRP antibody was purchased from Agilent (P0399). Fixation of microscopy samples was performed by adding 100 μL 4% paraformaldehyde in Phosphate Buffering Solution (PBS)++ (Fresenius Kabi, Zeist, The Netherlands) (PBS containing 0.5 MgCl2 and 1 mM CaCl2) to a drained flow chamber or to a coverslip. No permeabilization step was performed, as all antibodies bind to extracellular epitopes. Blocking was done with 2% Bovine Serum Albumin (BSA) in PBS++. Antibodies were incubated for 1 hour at RT. After all fixation and staining steps, the sample was washed 3x with PBS++.

#### Western blots

The western blot protocol followed was the same as.^8^ HUVECs were grown on FN-coated 6-well culture plates and washed twice with PBS++ (PBS + 0.5 MgCl_2_ and 1 mM CaCl_2_). For sample lysis, NP40 lysis buffer (50 mM TrisHCl, 100 mM NaCl, 10 mM MgCl_2_, 1% NP40 and 10% glycerol, pH7.4) with 1:500 protease inhibitor was used. Protein samples were centrifuged at 14.000 xG at RT for 10 minutes and resuspended in SDS-sample buffer containing 4% β-mecapto-ethanol. Samples were boiled at 95°C for 3 minutes to denature proteins and separated on a 4–12% NuPage Bis-Tris gel (Invitrogen, NP0322BOX). Proteins were transferred using an iBlot^2^ Gel Transfer device (Invitrogen, IB21001) for 7 minutes to a nitrocellulose membrane (Invitrogen, IB23001). Then, membranes were blocked with a 5% milk solution in tris-buffered saline with Tween 20 (TBST) at RT for 30 minutes. Primary antibodies were incubated overnight at 4 degrees in TBST. Secondary HRP antibodies were incubated at RT for 1 hour, after which a Pierce ECL Western Blotting Substrate kit (Themo Scientific, 32106) was used according to manufacturer’s instructions. After each blocking and staining step, the membranes were washed with TBST 3x minutes. Western blots were developed using an Amersham imager 600.

#### Flow cytometry

For flow cytometry experiments, 250.000 HUVECs per well were seeded in 12-well plates and grown to confluency in 2 days. TNFα was added 24 hours before the experiment. Cells were washed with PBS++ once, before adding 0.5 mL accutase (Sigma, A6964) for 7 minutes at 37°C and 5% CO_2_. 2 mL FACS buffer (PBS ++ with 0.5% BSA and 0.2 mM EDTA) was added to each well and cells were centrifuged for 5 min at 300 xG. The cell pellet was resuspended in ice-cold 1 mL FACS buffer and Near-IR Life-dead marker (Invitrogen, L10119A) was added 1:1000 and incubated for 30 min on ice. Cells were then centrifuged for 5 min at 300 xG at 4°C. 100.000 HUVECs of each condition were put into a well of 96-well plate for staining. Antibodies were incubated for 20 min on ice, after which cells were washed twice with ice-cold FACS buffer. Flow cytometry was performed on a Sony SP6800 Spectral analyzer. Single color controls were measured first, and their emission spectra were measured with a 488nm and 405nm/638 nm laser. These spectra were used for unmixing of multicolour samples using build-in software of the Spectral analyzer. The unmixed data was exported into and analyzed in FlowJo (Version 10). First, a population of non-doublet cells was gated. In this gate, only cells negative for the life-dead marker were gated to select only living cells. In this population, ICAM-1 surface expression was measured. A measurement of unstained cells was subtracted from the data.

#### Scanning electron microscopy

12 mm coverslips were coated with FN and put in a 24-well plate. 100.000 HUVECs were seeded in each well and grown to confluency in 2 days. TNFα was added 24 hours before the experiment. Cells were fixated in a 4% PFA and 2% glutaraldehyde in PBS++ solution for 1 hour. Afterwards, cells were dehydrated with increasing (50%, 60%, 70%, 80%, 90% to 96%) ethanol concentrations of 20 minutes each and submerged in hexamethyldisilane (Sigma-Aldrich) for 30 min. Next, samples were mounted on aluminum SEM stubs with Leit-C carbon cement (PLANO GmbH). A Leica EM ACE600 (Leica Microsystems) was used for sputter-coating with a 4 nm-thick platinum-palladium layer. Imaging was done on a Zeiss Sigma 300 SEM (Zeiss) at 2.00 or 3.00 kV, with magnifications ranging from 2.000x to 10.000x.

#### Neutrophil isolation

A protocol also described earlier by us was performed to isolate primary neutrophils.^8^ Blood was processed within 2 hours after donation. Whole blood was diluted 1:1 with 5% TNC in PBS and pipetted on 12.5 mL Percoll (1.076 g/ml). Next, a 20-minute centrifugation (Rotina 420R) at 800xg with a slow start and no brake was performed on the diluted blood, resulting in a separate erythrocyte/neutrophil pallet and a peripheral blood mononuclear (PBMC) ring. All except the erythrocyte/neutrophil pallet was then removed, and the pallet was resuspended in 45 mL ice-cold erythrocyte lysis buffer (155 mM NH4Cl, 10 mM KHCO3, 0.1 mM EDTA, pH7.4 in Milli-Q (Gibco, A1283-01)). Lysis was performed twice for 15 min on ice, with a 5 min 450xg centrifugation step after each lysis. Cells were then washed once with ice-cold PBS, followed again by a centrifugation if 5 min at 450xg. Cells were then resuspended in HEPES+ (20 mM HEPES, 132 mM NaCl, 6 mM KCl, 1 mM CaCl2, 1 mM MgSO4, 1.2 mM K2HPO4, 5 mM glucose (All Sigma-Aldrich), and 0.4% (w/v) human serum albumin (Sanquin Reagents), pH7.4 at RT at 1 million neutrophils / mL. A 20-min heat-activation at 37ᵒC was performed before using neutrophils in an experiment. Leukocyte counts were determined using a cell counter (Casey).

#### Neutrophil flow assay

30.000 HUVECs per lane were seeded in respectively FN- or collagen-coated Ibidi μ-slides VI^0.4^ (Ibidi, 80606-90) and grown for 48 hours, refreshing the medium twice each day. TNF-α treatment (10 ng/mL) was performed 24 hours before the experiment, when the endothelial cells were grown into a confluent monolayer. For tracking of leukocytes, cells were stained with Vybrant™ DiD Cell-labelling solution (Invitrogen, V22887) or Vybrant™ DiO Cell-labelling solution (Invitrogen, V22886) (both 1:6000) for 20 minutes at 37ᵒC in a concentration of 1–2 mil/mL neutrophils. Stained neutrophils were centrifuged for 3 minutes at 300xg at RT to wash away residual labelling solution and resuspended in fresh HEPES+ (100 mL HEPES + 100 mg D-Glucose + 2.5 mL Albuman + 100 μL 1M CaCl_2_). After a 20-minute recovery period, leukocytes were used for experiments. The Ibidi flow chamber containing the endothelial cells was connected to a perfusion system and underwent shear flow of 0.5 mL/min (0.8 dyne/cm^2^) for 2 minutes before injecting 700.000 leukocytes into the tubing system. Flow assays were imaged using an Axiovert 200 M widefield microscope, using a 10x NA 0.30 DIC Air objective (Zeiss). Fluorescent excitation light was provided by a HXP 120 C light source at 100% intensity and a TL Halogen Lamp at 6.06 V for transmitted light. Signal was detected with an AxioCam ICc 3 (Zeiss) camera. For the DIC channel, an exposure of 32 ms was used. For DiO-stained leukocytes, a 450–490 excitation filter, a 495 beam splitter, and a 500–550 emission filter were used with an exposure of 1900 ms. For DiD-stained leukocytes, a 625–655 excitation filter, a 660 nm beam splitter, and a 665–715 emission filter were used with an exposure of 1400 ms. To analyze leukocyte crawling dynamics and diapedesis locations Images were taken every 5 seconds for 15 minutes in two positions in the middle of the ibidi flow chamber lane. Immediately after acquiring the time-lapse, a tile-scan of 4x6 frames was collected to quantify total adhesion and transmigration numbers. Images were taken using Zeiss using Zen Blue software. The tile-scan was stitched using Zen Black software, using the DIC channel for stitching. Neutrophil adhesion, crawling dynamics and diapedesis efficacy were calculated in Imaris, as described in previous research.^8^ The generation of random spots in FIJI (v1.52p),^45^ and measurements of distance between neutrophils of both waves in Imaris was based on methods described before . First, in FIJI a number random spots, equal to the number of neutrophils measured in the second wave, was generated in the phase-contrast channel. In Imaris, a surface rendering was created of the neutrophils of the first wave. Next, a spot analysis was performed on both the randomly generated points and the neutrophils of the second wave. Using spot-surface measurements, Imaris calculated the distance of each spot (random spot/neutrophil of second wave) to the nearest surface (neutrophil of first spot). Negative distances mean that the spot was found inside the surface.

#### Lattice light sheet microscopy

Samples were imaged at the lattice light sheet microscope at the Advanced Imaging Center (AIC) at the Janelia Research Center of the Howard Hughes Medical Institute (HHMI).^26^ On 5 mm round coverslips (Warner Instruments, C5-5R) coated with FN, a 1:1 mosaic mixture of HUVECs stably expressing mNeonGreen-CaaX or mScarletI-CaaX was seeded and grown into a monolayer. Alternatively, cells stably expressing ICAM-1-GFP and LifeAct-mCherry were seeded. After a 24-hour TNF-α treatment, imaging was done in HEPES+ medium with temperature at 37°C and 5% CO_2_. For the CAAX-expressing HUVECS only, neutrophils were isolated as described above, and stained with Cell Tracker Red (Invitrogen, C34552). After staining, neutrophils were washed and centrifuged at 400xg for 3 min. Neutrophils were added on top of the coverslip, in between the detection (Nikon, CFI Apo LWD 25x, 1.1 NA) and excitation (Special Optics, 0.65 NA, 3.74 mm WD) objectives and incubated for a maximum of 30 minutes. For illumination, diode lasers of 488, 560 and 642 nm (MPB Communications) were used at 30% acousto-optic tunable filter (AOTF) transmittance and 50 mW initial box power. Fluorescence was detected using a Semrock FF01 – 446/523/600/677 quad bandpass filter and a sCMOS camera (Hamamatsu Orca Flash 4.0 v2). The exposure time was set on 20 ms with 50% AOTF transmittance and Z-step size was set on 0.211 μm. Time-lapse videos were recorded with an interval of about 7.5 seconds. Point-spread function was corrected in each channel using 200 nm tetraspeck beads (Invitrogen, T7280).

#### Creating stable KTR cell lines

To create stable cell lines that expressed the KTRs, a PiggyBac transposon transfection method was used.^46^ On approximately 3 million BOECs, diluted in 300 μL Opti-MEM (Gibco, 31985062) 6 μg ERK-AKT KTR or JNK-P38 KTR plasmid was microporated together with 6 μg transposase plasmid. After two days of culturing, a puromycin (1.5 μg/mL, 3 days) or blasticidin (2 μg/mL, 7 days) selection was performed for the ERK-AKT KTR and JNK-P38 KTR BOECs respectively. After selection, we sorted only the JNK-P38 KTR BOECs with the 25% lowest KTR expression, since it was shown previously that the JNKKTR sensor has higher dynamic range when its expression is low.

#### Kinase translocation reporter experiments

KTR imaging was performed at a Zeiss LSM 980 with Airyscan 2 module was used for confocal imaging of fixed samples, using a Plan-Apochromat 25x NA 0.8× water objective (Zeiss, 420852-9871-000) and pixel size of 0.33 × 0.33 micron. To make timelapses, we imaged at two positions every 30 seconds for the neutrophil experiments, for a total of 15 minutes, adding 100.000 neutrophils at t = 2.5 min. Before each experiment, cells were starved by keeping them in endothelial basal medium for 4 hours. For the TNFα-addition experiments, we imaged at two positions every 30 seconds for a total of 40 minutes, adding 10 ng/mL TNFα at t = 20 min. For the ERK-AKT KTR BOECs, we imaged mNeonGreen using a 488 nm laser with 0.3% laser power, mTurquoise2 with a 445 nm laser with 0.18% laser power and mScarletI using a 568 nm laser with 0.06% laser power. For the JNK-P38 KTR BOECs, we imaged mCherrry using a 568 nm laser with 0.05% laser power, mKO with a 445 nm laser with 0.3% laser power and iRFP670 using a 647 nm laser with 1% laser power. For the neutrophil crawling experiments, we added a widefield channel to be captured simultaneously with the mNeonGreen or mCherry channels in the ERK-AKT KTR and JNK-P38 KTR BOECs respectively. Data analysis done on KTR timelapse was heavily inspired and slightly adapted from earlier research.^25^ Pre-processing of the raw timelapse data was performed using FIJI (Methods S1). Here, all images were split into channels, and the nuclear channel underwent a background subtraction step (rolling ball, 50 pixels radius) to allow for better nuclear segmentation. Finally, the images were renamed to allow recognition by a CellProfiler pipeline (v3.0.0).^47^ The CellProfiler pipelines for the ERK-AKT KTR and JNK-P38 KTR BOECs were then used. First in this pipeline, nuclei were detected based on a 30–100 pixel diameter based on the nuclear marker, using a global manual threshold strategy of 0.1. These nuclei were then used as seeds to segment cells, segmenting a ring of 10 pixels around each nuclei, using a global manual threshold strategy of 0.08. Cytoplasmic regions were determined by subtracting the segmented cell area by the segmented nuclear area. Then, intensity of the nuclei and cytoplasmic regions was measured in both KTR channels at each timepoint. Nuclei and cytoplasmic regions were tracked by an overlap tracking algorithm, allowing a maximum pixel distance of 3 pixels. The exported data from this file was further processed in RStudio (v1.2.5001), using a script that cleans the data and extracts the C/N ratio of each cell over time into a csv file (Methods S2). This csv file was finally uploaded in PlotTwist to visualize the data in graphs.^48^ C/N data was normalized to all frames before addition of either TNFα or neutrophils. These data were the baseline and their value was subtracted from all timepoints, resulting in a relative kinase activity parameter which could be displayed in the graphs.

#### Measuring crosstalk between channels

To measure the crosstalk between the 3 FPs for each BOEC cell line, all FPs were transfected via microporation separately. For ERK-AKT KTR, mNeonGreen, mTurquoise2 and 3x-NLS mScarletI were transfected separately. For JNK-P38 KTR, MKO-MK2, JNK-KTR-mCherry and NLS-iRFP670 were transfected separately. Then using the exact same imaging settings that were used for the KTR experiments, fluorescent signal was measured in each channel for every FP, in the same cell. By measuring the fluorescent intensity of the brightest cell, using the same ROI for each image, one can calculate the crosstalk of each FP into all other channels, normalized to its fluorescence in its normal channel.

#### Clustering analysis

Clustering analysis was performed in PlotTwist.^48^ Both the control and neutrophil data were uploaded in the app together, and clustering was done based on Manhattan distance, using a Ward.D2 linkage method. Clustering was done based on two clusters. X-axis data was binned in pairs of 2 to smooth out the data, and the timepoints before neutrophil addition were excluded from the clustering algorithm. The contribution of each cluster in each condition was calculated automatically by PlotTwist.^48^

#### Widefield imaging and cell segmentation

Widefield imaging was performed at a Ti2 Microscope from Nikon, using a 20x air objective (Plan Apo λ 20x). Control and JNK inhibited cells, stained for nuclei, ICAM-1 and VE-cadherin, were imaged using the blue, red and far-red channels respectively. For the blue channel, an exposure of 200 ms with an excitation wavelength of 365 nm, combined with a 477/60 emission filter was used. For the red channel, an exposure of 200 ms with an excitation wavelength of 580 nm, combined with a 563/40 emission filter. Finally, for the far-red channel, an exposure of 700 nm was used in combination with a 692/40 emission filter. Images were analyzed using the Cells module in Imaris version 9.7.2. Cells were initially detected based on nuclear staining, using a 5 μm size to detect nuclei. The nuclei were filtered based on voxel number, with nuclei containing less than 173 voxels being filtered out. Next, cell outlines were detected via a watershed method from the nuclei and using the VE-cadherin stain. Membrane detail was set on 1.06 μm and cells with a diameter under 10.62 μm were filtered out. Finally, all cells that were not fully in frame were filtered out of the dataset. From this cell segmentation pipeline, we extracted cell area, cell sphericity and cell mean ICAM-1 fluorescent intensity. MARCKSL1 imaging was performed at a Ti2 Microscope from Nikon with a 60x objective (Apo TIRF 60x Oil DIC N2). HUVECs, stained for nuclei, F-actin, MARCKSL1 and ICAM-1 were imaged in the blue, green, red and far-red channels respectively. For the blue channel, an exposure of 500 ms with an excitation wavelength of 365 nm, combined with a 477/60 emission filter was used. For the green channel, an exposure of 300 ms with an excitation wavelength of 488 nm, combined with a 523/40 emission filter. For the red channel, an exposure of 300 ms with an excitation wavelength of 580 nm, combined with a 563/40 emission filter. Finally, for the far-red channel, an exposure of 30 nm with an excitation wavelength of 644 nm was used in combination with a 692/40 emission filter.

#### Fixed confocal imaging and filopodia quantifications

A Zeiss LSM 980 with Airyscan 2 module was used for detailed high-resolution confocal imaging of fixed samples, using a Plan-Apochromat 40x NA 1.3 oil DIC objective (Zeiss, 420762-9800-000) and a voxel size of 0.08 × 0.08 × 0.17 μm to capture Z-stacks. For all images, Multiplex SR-8Y settings were used and a GaAsP-PMT detector was used as a detector. Hoechst was excited using a 405 nm laser with 4% laser power. GFP was excited using a 488 laser with a laser power of 0.9%. Texas Red Phalloidin was excited with a 561 nm laser using 1.5% laser power. Alexa Fluor 647 was excited with a 639 nm laser with 1.8% laser power. To quantify filopodia density, a spot analysis on the ICAM-1 channel was performed in Imaris version 9.7.2. The estimated diameter used in this spot analysis was 0.6 μm. Spots were manually filtered based on quality, ensuring only filopodia and no background was measured. Here, we excluded junctional ICAM-1, either by negatively filtering using the VE-cadherin channel if there was one, or manually if there was not. Then, the total number of spots/filopodia was divided by the total surface imaged to create a filopodia/μm^2^ parameter. In the overexpression experiments, we calculated this parameter for both the transduced and non-transduced ECs separately by filtering spots based on the GFP-intensity at the same spot.

#### FRAP assay

ICAM-1-GFP was microporated into HUVECs, after which 150.000 HUVECs were seeded onto a 25 mm glass cover. Cells were cultured for 2 days, growing them into a monolayer. TNFα was added 24 hours before imaging to mimic inflammation. Imaging was done on a Zeiss LSM 980 Airyscan microscope, imaging 10 minutes total every 5 seconds. After the second frame, a 102 μm^2^ square was bleached for 30 iterations with a 100% 488 nm laser. Fluorescent signal was measured in the bleached square across the duration of the video and normalized to the intensity of the first frame.

### Quantification and statistical analysis

Data are presented as either means or medians + SD, indicated for each graph. For neutrophil quantifications, comparisons between two groups were performed by a t-test and comparisons between multiple groups were performed by One-way paired ANOVAs, pairing data of a single donor. For timelapse data, the 95% confidence interval was shown in the graph as a ribbon and data was considered significant if the 95% confidence intervals did not overlap. For other experiments, a Student’s *t* test or One-way ANOVA was performed, indicating which conditions were compared. A two-tailed p value of <0.05 was considered significant. For microscopy images, representative images are shown.

## Data Availability

• All western blot, flow cytometry and microscopy data reported in this paper will be shared by the lead contact upon request. • All original code for the KTR analyses is added as supplementary information. • Any additional information required to reanalyze the data reported in this paper is available upon request from the lead contact.
