# Supplementary material for: Primary adhered neutrophils increase JNK1-MARCKSL1-mediated filopodia to promote secondary neutrophil transmigration
Source: iScience. 2023 Jul 17;26(8):107406. doi: 10.1016/j.isci.2023.107406 (PMC10407253; doi:10.1016/j.isci.2023.107406)
Supplement: Document S1. Figure S1 and Table S1 [file mmc1.pdf]

iScience, Volume *26*

Supplemental information

Primary adhered neutrophils increase JNK1-MARCKSL1-mediated filopodia to promote secondary neutrophil transmigration

Max Laurens Bastiaan Grönloh, Janine Johanna Geertruida Arts, Eike Karin Mahlandt, Martijn A. Nolte, Joachim Goedhart, and Jaap Diederik van Buul

Figure S1. Generating KTR sensors in BOECs

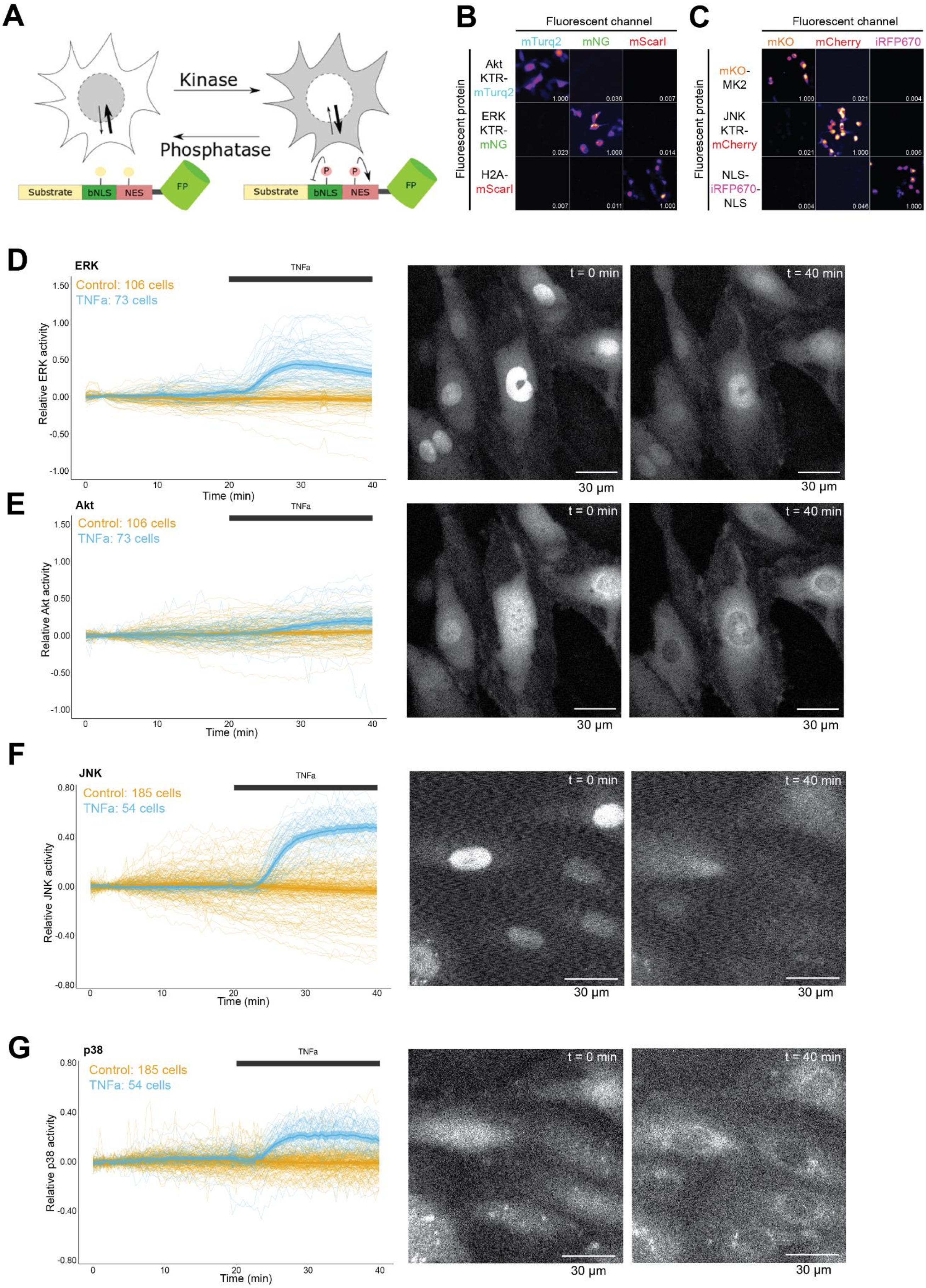

Figure S1: *Characterizing KTRs in endothelial cells,* related to Figure 3.

(A) Schematic overview of KTR sensors mode of action. Each KTR consists of an Fluorescent Protein (FP) coupled to a kinase specific substrate, which will be phosphorylated when the respective kinase is activated. Consequently, phosphorylation sites on a Nuclear Localization Signal (NLS) and Nuclear Export Signal (NES) will also be phosphorylated. This results in activating of the NES and inhibition of the NLS, and ensures the KTR will localize from the nucleus to the cytoplasm. Phosphatase activity will have the opposite effect. **(B)** Fluorescent images of BOECs overexpressing mNeonGreen, mTurquoise1 or 3x-NLS-mScarletI, imaged in the emission channels of each of these proteins. The number in the bottom right of each image displays the proportion of fluorescent signal of each FP in each emission channel. **(C)** Fluorescent images of BOECs overexpressing JNK-KTR-mCherry, MKO-MK2 or NLS-iRFP670, imaged in the emission channels of each of these proteins. The number in the bottom right of each image displays the proportion of fluorescent signal of each FP in each emission channel. **(D)** A time-series plot of ERK activation, measured by cytoplasmic/nuclear intensity of the ERK-KTR. Fluorescent images of the experiment at 0 and 40 minutes are displayed. Scale bar, 30 µm. **(E)** A time-series plot of Akt activation, measured by cytoplasmic/nuclear intensity of the Akt-KTR. Fluorescent images of the experiment at 0 and 40 minutes are displayed. Scale bar, 30 µm. **(D-E)** BOECs starved 4 hours before imaging. Control cells (orange, 106 cells) and cells with TNFα added at 20 minutes (blue, 73 cells) are displayed together. The darker and thicker line displays the mean of each condition, together with a 95% confidence interval. Data originate from 3 biological replicates. **(F)** A time-series plot of JNK activation, measured by cytoplasmic/nuclear intensity of the JNK-KTR. Fluorescent images of the experiment at 0 and 40 minutes are displayed. Scale bar, 30 µm. **(G)** A time-series plot of p38 activation, measured by cytoplasmic/nuclear intensity of the p38-KTR. Fluorescent images of the experiment at 0 and 40 minutes are displayed. Scale bar, 30 µm. **(F-G)** BOECs starved 4 hours before imaging. Control cells (orange, 185 cells) and cells with TNFα added at 20 minutes (blue, 54 cells) are displayed together. The darker and thicker line displays the mean of each condition, together with a 95% confidence interval. Data originate from 3 biological replicates.

Table S1, related to STAR methods. *All primers and siRNAs used in this study.*

No	Sequence	Use
PCR		
1<br>2	GAGCCTCTAGACAGCGCTCAATGGTG<br>GCCTAGCCTAATGTTAACGATCACTGC	GFP-JNK1
1<br>3	GAGCCTCTAGACAGCGCTCAATGGTG<br>GGCTAGCTTAATGTTAACGATCAGTTA	GFP-JNK2
Gibson cloning		
4<br>5	GAGCCTCTACAGAGCGCTCAATGGTGAGCAAGGGCGAG<br>GGCTAGCTTAATGTTAACGATCACTGCTGCACCTGTGC	pLV-GFP-JNK1
6<br>7	GAGCCTCTAGACAGCGCTCAATGGTGAGCAAGGGCGAG<br>GGCTAGCTTAATGTTAACGATCAGTTATCTAGATCCGGTGGATC	pLV-GFP-JNK2
SiRNAs		
8<br>*9*<br>*10*<br>*11*	GCGCUGAGCAGAAUGAGUA<br>GAACGGAACAGAUGAGGCA<br>UAGUGCAGCCUCAGAAGAA<br>GAGAGUGGCCCUACACCAG	MARCKSL1

Methods S1: FIJI macros for KTR pre-processing, related to Figure 3

ERK-AKT KTR without widefield channel

```
// Macro to prepare Czi files of KTR data for Cell profiler.

// You need 4 channels, of which the 3rd is the nuclear channel.

// Open all 3 series


dir1 = getDirectory("Choose Source Directory");      //Select the import folder

dir2 = getDirectory("Choose Destination Directory");  //Select the export folder

list = getFileList(dir1);                                              //list all files in import
folder

setBatchMode(true);

for (i=0; i<list.length; i++) {

   index_filmpje ="Filmpje_" + i + "_";

   showProgress(i+1, list.length);

   open(dir1+list[i]);

         filename = dir1 + list[i];                                    //process all files in the
folder


         imgArray = newArray(nImages);

         for (j=0; j<nImages; j++) {

                  selectImage(j+1);

                  imgArray[j] = getImageID();                          //proces all images in
each file

          }


         for (l=0; l< imgArray.length; l++) {

                  index_position = "Position_" + l;

                  selectImage(imgArray[l]);

                           imagename = getTitle();

                           imagename = replace(imagename, ".czi", "");

                           rename(imagename);

                           run("Split Channels");                             //split channels
```

```
			selectWindow("C1-"+ imagename);
			rename("Nucleus-"+ imagename + index_position + index_filmpje);
			run("Subtract Background...", "rolling=50 stack");
			run("Image Sequence... ", "format=TIFF save=dir2");
			close();
		//select nuclear channel and subtract BG and save C1 as image sequence


			selectWindow("C2-"+ imagename);
			rename("ERK-"+ imagename + index_position + index_filmpje);
			run("Image Sequence... ", "format=TIFF save=dir2");
			close();
		//Sace C2 as image sequence


			selectWindow("C3-"+ imagename);
			rename("Akt-"+ imagename + index_position + index_filmpje);
			run("Image Sequence... ", "format=TIFF save=dir2");
			close();
		//Sace C3 as image sequence


		}
	}
```

ERK-AKT KTR with widefield channel

// Macro to prepare Czi files of KTR data for Cell profiler.

// You need 4 channels, of which the 3rd is the nuclear channel.

// Open all 3 series

```
dir1 = getDirectory("Choose Source Directory");       //Select the import folder
dir2 = getDirectory("Choose Destination Directory");  //Select the export folder
list = getFileList(dir1);                                            //list all files in import
folder
setBatchMode(true);
for (i=0; i<list.length; i++) {
   index_filmpje ="Filmpje_" + i + "_";
   showProgress(i+1, list.length);
   open(dir1+list[i]);
        filename = dir1 + list[i];                                   //process all files
in the folder

        imgArray = newArray(nImages);
        for (j=0; j<nImages; j++) {
                selectImage(j+1);
                imgArray[j] = getImageID();                          //proces all images in
each file
         }

        for (l=0; l< imgArray.length; l++) {
                index_position = "Position_" + l;
                selectImage(imgArray[l]);
                        imagename = getTitle();
                        imagename = replace(imagename, ".czi", "");
                        rename(imagename);
                        run("Split Channels");                       //split channels

                        selectWindow("C1-"+ imagename);              //C1 = nucleus
```

```
                    rename("Nucleus-"+ imagename + index_position + index_filmpje);
                    run("Subtract Background...", "rolling=50 stack");
                    run("Image Sequence... ", "format=TIFF save=dir2");
                    close();
        //select nuclear channel and subtract BG and save as image sequence)

                    selectWindow("C2-"+ imagename);     //C2 = ERK
                    rename("ERK-"+ imagename + index_position + index_filmpje);
                    run("Image Sequence... ", "format=TIFF save=dir2");
                    close();

                    selectWindow("C3-"+ imagename);     //C3 = brightfield
                    rename("Brightfield-"+ imagename + index_position + index_filmpje);
                    run("Image Sequence... ", "format=TIFF save=dir2");
                    close();

                    selectWindow("C4-"+ imagename);     //C4 = Akt
                    rename("Akt-"+ imagename + index_position + index_filmpje);
                    run("Image Sequence... ", "format=TIFF save=dir2");
                    close();
        }
}
```

JNK-P38 KTR without widefield channel

```
// Macro to prepare Czi files of KTR data for Cell profiler.

// You need 4 channels, of which the the first is the nuclear channel.

// Open all 3 series


dir1 = getDirectory("Choose Source Directory");       //Select the import folder

dir2 = getDirectory("Choose Destination Directory");  //Select the export folder

list = getFileList(dir1);                                             //list all files in import
folder

setBatchMode(true);

for (i=0; i<list.length; i++) {

    index_filmpje ="Filmpje_" + i + "_";

    showProgress(i+1, list.length);

    open(dir1+list[i]);

        filename = dir1 + list[i];                                          //process all files
in the folder


        imgArray = newArray(nImages);

        for (j=0; j<nImages; j++) {

                selectImage(j+1);

                imgArray[j] = getImageID();                             //proces all images in
each file

        }


        for (l=0; l< imgArray.length; l++) {

                index_position = "Position_" + l;

                selectImage(imgArray[l]);

                        imagename = getTitle();

                        imagename = replace(imagename, ".czi", "");

                        rename(imagename);

                        run("Split Channels");                              //split channels


                        selectWindow("C1-"+ imagename);             //C1 = nucleus
```

```
            rename("Nucleus-"+ imagename + index_position + index_filmpje);
            run("Subtract Background...", "rolling=50 stack");
            run("Image Sequence... ", "format=TIFF save=dir2");
            close();
    //select nuclear channel and subtract BG and save as image sequence)


            selectWindow("C2-"+ imagename);        //C2 = JNK
            rename("JNK-"+ imagename + index_position + index_filmpje);
            run("Image Sequence... ", "format=TIFF save=dir2");
            close();


            selectWindow("C3-"+ imagename);        //C3 = p38
            rename("p38-"+ imagename + index_position + index_filmpje);
            run("Image Sequence... ", "format=TIFF save=dir2");
            close();

    }
}
```

JNK-P38 KTR with widefield channel

// Macro to prepare Czi files of KTR data for Cell profiler.

// You need 4 channels, of which the the first is the nuclear channel.

// Open all 3 series

```
dir1 = getDirectory("Choose Source Directory");       //Select the import folder
dir2 = getDirectory("Choose Destination Directory");  //Select the export folder
list = getFileList(dir1);                                               //list all files in import
folder
setBatchMode(true);
for (i=0; i<list.length; i++) {
   index_filmpje ="Filmpje_" + i + "_";
   showProgress(i+1, list.length);
   open(dir1+list[i]);
         filename = dir1 + list[i];                                      //process all files
in the folder

         imgArray = newArray(nImages);
         for (j=0; j<nImages; j++) {
                 selectImage(j+1);
                 imgArray[j] = getImageID();                        //proces all images in
each file
          }

         for (l=0; l< imgArray.length; l++) {
                 index_position = "Position_" + l;
                 selectImage(imgArray[l]);
                         imagename = getTitle();
                         imagename = replace(imagename, ".czi", "");
                         rename(imagename);
                         run("Split Channels");                      //split channels

                         selectWindow("C1-"+ imagename);          //C1 = nucleus
```

```
			rename("Nucleus-"+ imagename + index_position + index_filmpje);
			run("Subtract Background...", "rolling=50 stack");
			run("Image Sequence... ", "format=TIFF save=dir2");
			close();
	//select nuclear channel and subtract BG and save as image sequence)

			selectWindow("C2-"+ imagename);		//C2 = JNK
			rename("JNK-"+ imagename + index_position + index_filmpje);
			run("Image Sequence... ", "format=TIFF save=dir2");
			close();

			selectWindow("C3-"+ imagename);		//C3 = brightfield
			rename("brightfield-"+ imagename + index_position + index_filmpje);
			run("Image Sequence... ", "format=TIFF save=dir2");
			close();

			selectWindow("C4-"+ imagename);		//C4 = p38
			rename("p38-"+ imagename + index_position + index_filmpje);
			run("Image Sequence... ", "format=TIFF save=dir2");
			close();
	}
}
```

Methods S2: R script KTR analysis, related to Figure 3

ERK-AKT KTR

```
remove.packages("rlang")
install.packages("rlang")
library(rlang)
library(dplyr)

require(dplyr)
require(ggplot2)
require(magrittr)
require(stats)
require(tidyr)
require(gridExtra)
require(stringr)
library(data.table)
require(base)
library(filesstrings)
library(readr)

#Create for loop for each csv
setwd("File location of input images")
path <- getwd()

time_points <- Number of timepoints
time_interval <- Interval in min
time_well <- 0
time_before_start <- 0
mulfac <- 10000
baseline <- Timepoint until which to calculate baseline
radius <- 1

### Font size for the axis and labels
```

```
fs <- 1.5

######## Function to generate 95%CI
get_CI_half_width <- function(x) {
  n <- length(x)
  z_t <- qt(1 - (1 - 0.95) / 2, df = n - 1)
  z_t * sd(x) / sqrt(n)
}
lower <- function(x) {
  mean(x) - get_CI_half_width(x)
}
upper <- function(x) {
  mean(x) + get_CI_half_width(x)
}
sem <- function(x){
  n <- length(x)
  sd(x, na.rm=TRUE)/sqrt(length(x[!is.na(x)]))
}
upper_sem <- function(x){
  mean(x) + sem(x)
}
lower_sem <- function(x){
  mean(x) - sem(x)
}
upper_sd <- function(x){
  mean(x) + sd(x)
}
lower_sd <- function(x){
  mean(x) - sd(x)
}

csv_list <- list.files(path, pattern="Nuclei.csv|Cytoplasm.csv")
```

```
ind <- 0
for (i in csv_list){
  ind <- ind +1
  df_temp = read.csv(i)
  # filename1 <- gsub(csv_list[ind], pattern="\\..*", replacement=".csv")
  # write.csv(df_temp, file=filename1, row.names = FALSE)
  filename <- gsub(csv_list[ind], pattern="\\..*", replacement="_filter.csv")

  #Select and rename columns of interest
  df_temp <- df_temp %>% select(Time= Metadata_Time, Area= AreaShape_Area,
Mean_Radius= AreaShape_MeanRadius, contains("MeanIntensity_"), contains("Math"),
Object_Label= TrackObjects_Label_3)
  names(df_temp) <- gsub("Intensity_MeanIntensity_", "Intensity_", names(df_temp))

  #Filter rows with NA in Object_label column, objects that have a very thin cytoplasm and
with very low/high fluorescence
  df_filtered <- df_temp %>%  filter(!is.na(Object_Label))
  #df_filtered <- df_filtered %>% group_by(Object_Label) %>% filter(mean(Mean_Radius) >
radius)
  df_filtered <- df_filtered %>% filter(Intensity_ERK>0.0010)
  df_filtered <- df_filtered %>% filter(Intensity_ERK<0.9999)
  df_filtered <- df_filtered %>% filter(Intensity_Akt>0.0010)
  df_filtered <- df_filtered %>% filter(Intensity_Akt<0.9999)

  #df_filtered <- df_filtered %>% filter(Area>100)

  #Find and select (=filter) the Objectlabels that are present at all timepoints and in different
frames
  df_filtered <- df_filtered %>% group_by(Object_Label) %>% add_tally() %>%
filter(n==time_points)
  #df_filtered <- df_filtered %>% group_by(Object_Label) %>%
filter(sum(Time)==(time_points*(time_points+1))/2)
  df_filtered <- df_filtered %>% mutate(Unique_Object_Label=(Object_Label))
  df_filtered$n = NULL
  df_filtered$Object_Label = NULL
```

```
  df_filtered[4:5]=round(df_filtered[4:5],5)
  write.csv(df_filtered,file=filename, row.names = FALSE)
}

Nuclei= read.csv(file="Nuclei_filter.csv", header=TRUE, sep=",")
Cytoplasm= read.csv(file="Cytoplasm_filter.csv", header=TRUE, sep=",")

### Specify ROI
x <- (colnames(Nuclei[0,2:(ncol(Nuclei)-1)]))
for (i2 in x){
  colnames(Nuclei)[colnames(Nuclei)==i2] <- paste(c("Nuclear", i2), collapse='_')
  colnames(Cytoplasm)[colnames(Cytoplasm)==i2] <- paste(c("Cytoplasmic", i2),
collapse='_')}
merged <- merge(Nuclei, Cytoplasm)

### Calculate CN ratios and time in minutes
merged <- merged %>% mutate(CN_Akt= Cytoplasmic_Intensity_Akt/Nuclear_Intensity_Akt)
merged <- merged %>% mutate(CN_ERK=
Cytoplasmic_Intensity_ERK/Nuclear_Intensity_ERK)
merged <- merged %>% mutate(Time_in_min=time_before_start + (Time-1)*time_interval)
merged$Time_in_min=round(merged$Time_in_min,1)

#Get normalized ratios
merged <-  merged %>% arrange(Time) %>% group_by(Unique_Object_Label)  %>%
arrange(Unique_Object_Label) %>%
        mutate(CN_Akt_norm=CN_Akt-mean(CN_Akt[1:baseline]),
CN_ERK_norm=CN_ERK-mean(CN_ERK[1:baseline]))

merged[5:6] <- merged[5:6] * mulfac
merged[9:10] <- merged[9:10] * mulfac
merged[3:ncol(merged)] <- round(merged[3:ncol(merged)],4)
write.csv(merged, "Nuclei+Cytoplasm.csv", row.names = FALSE)

################### Calculate mean and 95% CI
```

```
z <- paste(colnames(merged[,9:13]), collapse = ", ")

df_stats_summary <- merged %>% group_by(Time_in_min) %>% select(CN_Akt, CN_ERK, Time_in_min, CN_Akt_norm, CN_ERK_norm) %>% summarise_all(funs(mean, upper, lower, upper_sd, lower_sd))


###### Define basic plot layout

plt <- ggplot(merged, aes(x=Time_in_min)) + theme_light(base_size = 16) + xlab("time (min)") + theme(text = element_text(size=20), axis.text.x = element_text(size = rel(fs)),

                                                                                           axis.text.y = element_text(size = rel(fs)), axis.title.y = element_text(size = rel(fs)), axis.title.x = element_text(size = rel(fs)))


###### Define plot for raw data

plt1 <- plt   + ylab("CN ERK") + geom_line(data=merged, aes(x=Time_in_min, y=CN_ERK, group=Unique_Object_Label), color="grey", alpha=0.2) +

  geom_line(data = df_stats_summary, aes(x=Time_in_min, y=CN_ERK_mean), color="black", size=1.2) +

  geom_ribbon(data = df_stats_summary, aes(x=Time_in_min, ymin=CN_ERK_lower_sd, ymax=CN_ERK_upper_sd) ,fill="blue", alpha=0.1) +

  coord_cartesian(ylim = c(0.2, 1.5)) +

  NULL


plt3 <- plt   + ylab("CN Akt RB") + geom_line(aes(x=Time_in_min, y=CN_Akt, group=Unique_Object_Label), color="grey", alpha=0.2) +

  geom_line(data = df_stats_summary, aes(x=Time_in_min, y=CN_Akt_mean), color="black", size=1.2) +

  geom_ribbon(data = df_stats_summary, aes(x=Time_in_min, ymin=CN_Akt_lower_sd, ymax=CN_Akt_upper_sd) ,fill="blue", alpha=0.1) +

  coord_cartesian(ylim = c(0.2, 1.2)) +

  NULL


#### Define plot for normalized data

plt1n <- plt   + ylab("change in CN ERK")+ geom_line(data=merged, aes(x=Time_in_min, y=CN_ERK_norm,group=Unique_Object_Label), color="grey", alpha=0.2) +

  geom_line(data = df_stats_summary, aes(x=Time_in_min, y=CN_ERK_norm_mean), color="black", size=1.2) +
```

```
  geom_ribbon(data = df_stats_summary, aes(x=Time_in_min,
ymin=CN_ERK_norm_lower_sd, ymax=CN_ERK_norm_upper_sd) ,fill="blue", alpha=0.1) +
  coord_cartesian(ylim = c(-0.2, 0.8)) +
  NULL


plt3n <- plt   + ylab("change in CN Akt RB") + geom_line(aes(x=Time_in_min,
y=CN_Akt_norm,group=Unique_Object_Label), color="grey", alpha=0.2) +
  geom_line(data = df_stats_summary, aes(x=Time_in_min, y=CN_Akt_norm_mean),
color="black", size=1.2) +
  geom_ribbon(data = df_stats_summary, aes(x=Time_in_min,
ymin=CN_Akt_norm_lower_sd, ymax=CN_Akt_norm_upper_sd) ,fill="blue", alpha=0.1) +
  coord_cartesian(ylim = c(-0.3, 0.6)) +
  NULL


#### Arrange the plots and tables on a single page and plot ####
p1 <- grid.arrange(plt1, plt3, ncol=2)
p2 <- grid.arrange(plt1n, plt3n, ncol=2)


p1
png(file="not_norm_plots.png", width = 2048, height = 680, units = "px")
plot(p1)
dev.off()


p2
png(file="norm_plots 100.png", width = 2048, height = 680, units = "px")
plot(p2)
dev.off()


sumplot
png(file="Summary.png", width = 2048, height = 1000, units = "px")
plot(sumplot)
dev.off()
```

JNK-P38 KTR

remove.packages("rlang")

install.packages("rlang")

library(rlang)

library(dplyr)

require(dplyr)

require(ggplot2)

require(magrittr)

require(stats)

require(tidyr)

require(gridExtra)

require(stringr)

library(data.table)

require(base)

library(filesstrings)

library(readr)

#Create for loop for each csv

#Create for loop for each csv

setwd("**File location of input images**")

path <- getwd()

time_points <- **Number of timepoints**

time_interval <- **Interval in min**

time_well <- 0

time_before_start <- 0

mulfac <- 10000

baseline <- **Timepoint until which to calculate baseline**

radius <- 1

Font size for the axis and labels

```
fs <- 1.5

######## Function to generate 95%CI
get_CI_half_width <- function(x) {
  n <- length(x)
  z_t <- qt(1 - (1 - 0.95) / 2, df = n - 1)
  z_t * sd(x) / sqrt(n)
}
lower <- function(x) {
  mean(x) - get_CI_half_width(x)
}
upper <- function(x) {
  mean(x) + get_CI_half_width(x)
}
sem <- function(x){
  n <- length(x)
  sd(x, na.rm=TRUE)/sqrt(length(x[!is.na(x)]))
}
upper_sem <- function(x){
  mean(x) + sem(x)
}
lower_sem <- function(x){
  mean(x) - sem(x)
}
upper_sd <- function(x){
  mean(x) + sd(x)
}
lower_sd <- function(x){
  mean(x) - sd(x)
}

csv_list <- list.files(path, pattern="Nuclei.csv|Cytoplasm.csv")
```

```
ind <- 0
for (i in csv_list){
  ind <- ind +1
  df_temp = read.csv(i)
  # filename1 <- gsub(csv_list[ind], pattern="\\..*", replacement=".csv")
  # write.csv(df_temp, file=filename1, row.names = FALSE)
  filename <- gsub(csv_list[ind], pattern="\\..*", replacement="_filter.csv")

  #Select and rename columns of interest
  df_temp <- df_temp %>% select(Time= Metadata_Time, Area= AreaShape_Area, Mean_Radius= AreaShape_MeanRadius, contains("MeanIntensity_"), contains("Math"), Object_Label= TrackObjects_Label_3)
  names(df_temp) <- gsub("Intensity_MeanIntensity_", "Intensity_", names(df_temp))

  #Filter rows with NA in Object_label column, objects that have a very thin cytoplasm and with very low/high fluorescence
  df_filtered <- df_temp %>%  filter(!is.na(Object_Label))
  #df_filtered <- df_filtered %>% group_by(Object_Label) %>% filter(mean(Mean_Radius) > radius)
  df_filtered <- df_filtered %>% filter(Intensity_JNK>0.0010)
  df_filtered <- df_filtered %>% filter(Intensity_JNK<0.9999)
  df_filtered <- df_filtered %>% filter(Intensity_p38>0.0010)
  df_filtered <- df_filtered %>% filter(Intensity_p38<0.9999)

  #df_filtered <- df_filtered %>% filter(Area>100)

  #Find and select (=filter) the Objectlabels that are present at all timepoints and in different frames
  df_filtered <- df_filtered %>% group_by(Object_Label) %>% add_tally() %>% filter(n==time_points)
  #df_filtered <- df_filtered %>% group_by(Object_Label) %>% filter(sum(Time)==(time_points*(time_points+1))/2)
  df_filtered <- df_filtered %>% mutate(Unique_Object_Label=(Object_Label))
  df_filtered$n = NULL
  df_filtered$Object_Label = NULL
```

```
  df_filtered[4:5]=round(df_filtered[4:5],5)
  write.csv(df_filtered,file=filename, row.names = FALSE)
}

Nuclei= read.csv(file="Nuclei_filter.csv", header=TRUE, sep=",")
Cytoplasm= read.csv(file="Cytoplasm_filter.csv", header=TRUE, sep=",")

### Specify ROI
x <- (colnames(Nuclei[0,2:(ncol(Nuclei)-1)]))
for (i2 in x){
  colnames(Nuclei)[colnames(Nuclei)==i2] <- paste(c("Nuclear", i2), collapse='_')
  colnames(Cytoplasm)[colnames(Cytoplasm)==i2] <- paste(c("Cytoplasmic", i2), 
collapse='_')}
merged <- merge(Nuclei, Cytoplasm)

### Calculate CN ratios and time in minutes
merged <- merged %>% mutate(CN_p38= 
Cytoplasmic_Intensity_p38/Nuclear_Intensity_p38)
merged <- merged %>% mutate(CN_JNK= 
Cytoplasmic_Intensity_JNK/Nuclear_Intensity_JNK)
merged <- merged %>% mutate(Time_in_min=time_before_start + (Time-1)*time_interval)
merged$Time_in_min=round(merged$Time_in_min,1)

#Get normalized ratios
merged <-  merged %>% arrange(Time) %>% group_by(Unique_Object_Label)  %>% 
arrange(Unique_Object_Label) %>%
        mutate(CN_p38_norm=CN_p38-mean(CN_p38[1:baseline]), 
CN_JNK_norm=CN_JNK-mean(CN_JNK[1:baseline]))

merged[5:6] <- merged[5:6] * mulfac
merged[9:10] <- merged[9:10] * mulfac
merged[3:ncol(merged)] <- round(merged[3:ncol(merged)],4)
write.csv(merged, "Nuclei+Cytoplasm.csv", row.names = FALSE)
```

```
################### Calculate mean and 95% CI

z <- paste(colnames(merged[,9:13]), collapse = ", ")

df_stats_summary <- merged %>% group_by(Time_in_min) %>% select(CN_p38, CN_JNK, Time_in_min, CN_p38_norm, CN_JNK_norm) %>% summarise_all(funs(mean, upper, lower, upper_sd, lower_sd))


###### Define basic plot layout

plt <- ggplot(merged, aes(x=Time_in_min)) + theme_light(base_size = 16) + xlab("time (min)") + theme(text = element_text(size=20), axis.text.x = element_text(size = rel(fs)),
                                                                                                         axis.text.y = element_text(size = rel(fs)), axis.title.y = element_text(size = rel(fs)), axis.title.x = element_text(size = rel(fs)))


###### Define plot for raw data

plt1 <- plt   + ylab("CN JNK") + geom_line(data=merged, aes(x=Time_in_min, y=CN_JNK, group=Unique_Object_Label), color="grey", alpha=0.2) +
  geom_line(data = df_stats_summary, aes(x=Time_in_min, y=CN_JNK_mean), color="black", size=1.2) +
  geom_ribbon(data = df_stats_summary, aes(x=Time_in_min, ymin=CN_JNK_lower_sd, ymax=CN_JNK_upper_sd) ,fill="blue", alpha=0.1) +
  coord_cartesian(ylim = c(0.2, 1.5)) +
  NULL


plt3 <- plt   + ylab("CN p38") + geom_line(aes(x=Time_in_min, y=CN_p38, group=Unique_Object_Label), color="grey", alpha=0.2) +
  geom_line(data = df_stats_summary, aes(x=Time_in_min, y=CN_p38_mean), color="black", size=1.2) +
  geom_ribbon(data = df_stats_summary, aes(x=Time_in_min, ymin=CN_p38_lower_sd, ymax=CN_p38_upper_sd) ,fill="blue", alpha=0.1) +
  coord_cartesian(ylim = c(0.2, 1.2)) +
  NULL


#### Define plot for normalized data

plt1n <- plt   + ylab("change in CN JNK")+ geom_line(data=merged, aes(x=Time_in_min, y=CN_JNK_norm,group=Unique_Object_Label), color="grey", alpha=0.2) +
  geom_line(data = df_stats_summary, aes(x=Time_in_min, y=CN_JNK_norm_mean), color="black", size=1.2) +
```

```
  geom_ribbon(data = df_stats_summary, aes(x=Time_in_min,
ymin=CN_JNK_norm_lower_sd, ymax=CN_JNK_norm_upper_sd) ,fill="blue", alpha=0.1) +
  coord_cartesian(ylim = c(-0.2, 0.8)) +
  NULL


plt3n <- plt   + ylab("change in CN p38") + geom_line(aes(x=Time_in_min,
y=CN_p38_norm,group=Unique_Object_Label), color="grey", alpha=0.2) +
  geom_line(data = df_stats_summary, aes(x=Time_in_min, y=CN_p38_norm_mean),
color="black", size=1.2) +
  geom_ribbon(data = df_stats_summary, aes(x=Time_in_min,
ymin=CN_p38_norm_lower_sd, ymax=CN_p38_norm_upper_sd) ,fill="blue", alpha=0.1) +
  coord_cartesian(ylim = c(-0.3, 0.6)) +
  NULL


#### Arrange the plots and tables on a single page and plot ####
p1 <- grid.arrange(plt1, plt3, ncol=2)
p2 <- grid.arrange(plt1n, plt3n, ncol=2)


p1
png(file="not_norm_plots.png", width = 2048, height = 680, units = "px")
plot(p1)
dev.off()


p2
png(file="norm_plots 100.png", width = 2048, height = 680, units = "px")
plot(p2)
dev.off()


sumplot
png(file="Summary.png", width = 2048, height = 1000, units = "px")
plot(sumplot)
dev.off()
```
